# A coevolution experiment between *Flavobacterium johnsoniae* and *Burkholderia thailandensis* reveals parallel mutations that reduce antibiotic susceptibility

**DOI:** 10.1099/mic.0.001267

**Published:** 2023-02-01

**Authors:** John L. Chodkowski, Ashley Shade

**Affiliations:** ^1^​ Department of Microbiology and Molecular Genetics, Michigan State University, East Lansing, MI 48824, USA; ^2^​ Department of Plant, Soil and Microbial Sciences, Michigan State University, East Lansing, MI 48824, USA; ^3^​ Program in Ecology, Evolution and Behavior, Michigan State University, East Lansing, MI 48824, USA; ^4^​ Univ Lyon, CNRS, INSA Lyon, Université Claude Bernard Lyon 1, Ecole Centrale de Lyon, Ampère, UMR5005, 69134, Ecully cedex, France

**Keywords:** *Burkholderia thailandensis *E264, *Flavobacterium johnsoniae *UW101, coevolution, TolC, efflux, thailandamide, interference competition

## Abstract

One interference mechanism of bacterial competition is the production of antibiotics. Bacteria exposed to antibiotics can resist antibiotic inhibition through intrinsic or acquired mechanisms. Here, we performed a coevolution experiment to understand the long-term consequences of antibiotic production and antibiotic susceptibility for two environmental bacterial strains. We grew five independent lines of the antibiotic-producing environmental strain, *Burkholderia thailandensis* E264, and the antibiotic-inhibited environmental strain, *Flavobacterium johnsoniae* UW101, together and separately on agar plates for 7.5 months (1.5 month incubations), transferring each line five times to new agar plates. We observed that the *F. johnsoniae* ancestor could tolerate the *B. thailandensis *-produced antibiotic through efflux mechanisms, but that the coevolved lines had reduced susceptibility. We then sequenced genomes from the coevolved and monoculture *F. johnsoniae* lines, and uncovered mutational ramifications for the long-term antibiotic exposure. The coevolved genomes from *F. johnsoniae* revealed four potential mutational signatures of reduced antibiotic susceptibility that were not observed in the evolved monoculture lines. Two mutations were found in *tolC*: one corresponding to a 33 bp deletion and the other corresponding to a nonsynonymous mutation. A third mutation was observed as a 1 bp insertion coding for a RagB/SusD nutrient uptake protein. The last mutation was a G83R nonsynonymous mutation in acetyl-coA carboxylayse carboxyltransferase subunit alpha (AccA). Deleting the 33 bp from *tolC* in the *F. johnsoniae* ancestor reduced antibiotic susceptibility, but not to the degree observed in coevolved lines. Furthermore, the *accA* mutation matched a previously described mutation conferring resistance to *B. thailandensis *-produced thailandamide. Analysis of *B. thailandensis* transposon mutants for thailandamide production revealed that thailandamide was bioactive against *F. johnsoniae,* but also suggested that additional *B. thailandensis *-produced antibiotics were involved in the inhibition of *F. johnsoniae *. This study reveals how multi-generational interspecies interactions, mediated through chemical exchange, can result in novel interaction-specific mutations, some of which may contribute to reductions in antibiotic susceptibility.

## Introduction

Cultivation-independent sequencing of environmental microbial DNA has revealed the prevalence of antibiotic resistance genes in pristine environments [1], indicating that antibiotics and their corresponding resistance mechanisms have long evolved in natural environments that predate their use in medicine [2]. For example, glycopeptide antibiotics and resistance mechanisms have been present in bacterial genomes for at least 150 million years [3]. Thus, it is expected that understanding the evolution of antibiotic resistance in ‘natural’ settings will provide insights into emerging mechanisms of antibiotic resistance that may be useful for addressing resistance in clinical settings [4].

Microbial antibiotic production in the environment is typically viewed through the lens of competition [5]. Bacteria produce antibiotics that interfere directly with competitors by inflicting cell damage [6]. The DNA blueprints for antibiotics are organized in biosynthetic gene clusters (BSGCs), in which locally proximal genes collectively encode the pathway for molecule production [7]. The activation of BSGCs is typically tied to stress regulation [8], suggesting that antibiotic production can be deployed as a survival strategy when conditions are not optimal for growth [9].

Bacteria can survive antibiotic exposure through the upregulation of intrinsic resistance mechanisms and can achieve antibiotic resistance through acquired mechanisms (e.g. mutation or horizontal gene transfer [10]). Multidrug efflux pumps are particularly interesting because they can provide both intrinsic and acquired resistance mechanisms [11]. For example, low levels of antibiotic exposure can upregulate intrinsic mechanisms of resistance, such as efflux pumps [12, 13]. The survival of bacteria against low levels of antibiotics can facilitate adaptive resistance [14]. This has been demonstrated by clinically relevant antibiotic resistance achieved via efflux pumps [15]. Therefore, studying multigenerational interactions between antibiotic-producing and antibiotic-inhibited environmental isolates may provide insights into the evolutionary dynamics driving antibiotic resistance.

We were interested in the competitive ability of two environmental strains*, Burkholderia thailandensis* and *Flavobacterium johnsoniae *. A previous study examined the competitive ability of *F. johnsoniae* against *B. thailandensis* through contact-dependent mechanisms involving the type VI secretion system [16]. Given the nature of *B. thailandensis* as a prolific secondary metabolite producer, including various antibiotics [17], and the various TolC efflux systems observed in *F. johnsoniae *, we wanted to expand the exploration of interspecies competition between these strains by focusing on contact-independent mechanisms. Preliminary results suggested that *F. johnsoniae* was susceptible to the *B. thailandensis *-produced antibiotic(s), but intrinsic mechanisms, such as efflux systems, allowed *F. johnsoniae* to tolerate the presence of the antibiotic(s) and permit growth on agar. We then tested whether these intrinsic mechanisms of antibiotic tolerance would pave the way for mutational acquisition in *F. johnsoniae* that would make it less susceptible to antibiotics.

We performed an agar-based experimental coevolution with *B. thailandensis* and *F. johnsoniae *. These strains were co-plated together and, in parallel, also plated in monocultures on M9 minimal medium agar plates containing 0.2 % glucose (M9 glucose; % v/v) for 7.5 months. *B. thailandensis* and *F. johnsoniae* were co-plated such that *B. thailandensis* antibiotic inhibition of *F. johnsoniae* could occur without intergrowth of the colonies. By comparing outcomes of the coevolved lines to the evolved monoculture lines, we asked: what are the genetic and phenotypic repercussions of coevolution and how consistent are they across independent, replicate lines? What are the genetic signatures of *F. johnsoniae* of reduced antibiotic susceptibility? What is the antibiotic produced by *B. thailandensis* that inhibits *F. johnsoniae *? We found that coevolved *F. johnsoniae* lines reduced susceptibility to the *B. thailandensis *-produced antibiotic while evolved monoculture lines remained susceptible. A 33 bp deletion in *tolC* and a nonsynonymous mutation in *accA* suggested two different paths to the evolution of reduced antibiotic susceptibility in *F. johnsoniae *. The ancestor *F. johnsoniae* strain became less susceptible after we deleted the *tolC* 33 bp region, but not to an equivalent level observed in the coevolved lines. This result suggests that multiple mutations may contribute to the antibiotic susceptibility. Although genomics from *B. thailandensis* did not further inform us as to the bioactive compound(s), a mutation in *F. johnsoniae* provided evidence that thailandamide, a previously described antibiotic that inhibits fatty acid synthesis [18], was among the bioactive compounds that inhibited *F. johnsoniae *. Our data also suggest that multiple antibiotics produced by *B. thailandensis* contributed to the observed inhibition of *F. johnsoniae *.

## Methods

### Extraction of *B. thailandensis* supernatant containing antibiotic activity

A freezer stock of *B. thailandensis* was plated on 50 % trypticase soy agar (TSA_50_) and grown for ~16 h at 27 °C. A loopful of lawn growth was transferred to 7 ml of M9 minimal salts 0.2 % glucose (M9 glucose; % v/v) medium to achieve an initial OD_590_ of ~0.2, as measured on an Evolution 60S UV–visible spectrophotometer (Thermo Scientific). The culture was incubated with shaking at 200 r.p.m. for ~16 h at 27 °C. The next day, 1 ml of culture was transferred to 50 ml fresh M9 glucose medium. The culture was incubated with shaking at 200 r.p.m. for 24 h at 27 °C. The culture was transferred to a falcon tube and centrifuged at 5000 r.p.m. for 20 min at 4 °C . The supernatant was removed, filtered with a 0.22 µM PES filter, and transferred to a separatory funnel. Ten millilitres of dichloromethane (DCM) was added to the separatory funnel. The separatory funnel was agitated three times and the DCM layer was removed. The addition, agitation and removal of DCM was repeated two more times. The collected DCM layer was dried under nitrogen gas. The dried DCM extracts were reconstituted in 1 ml of a 50 : 50 methanol : water (% v/v) mixture. This mixture was used for the efflux pump inhibitor experiment.

### Efflux pump inhibitor experiment

A freezer stock of *F. johnsoniae* was plated on TSA_50_ and grown for ~16 h at 27 °C. Five individual colonies were then inoculated in 5 ml of 50 % trypticase soy broth (TSB_50_) and incubated with shaking at 200 r.p.m. for ~16 h at 27 °C. The following day, 50 µl of culture was diluted into 4.95 ml of fresh TSB_50_ for each independent replicate. Then, 650 µl aliquots were dispensed in each of seven tubes for each independent replicate. Seven conditions were prepared across the seven tubes as follows: (1) *F. johnsoniae* culture control; (2) *F. johnsoniae* with DMSO control; (3) *F. johnsoniae* with 50 : 50 methanol : water (% v/v) control; (4) *F. johnsoniae* with DMSO+50 : 50 methanol : water (% v/v) control; (5) *F. johnsoniae* with daidzein+50 : 50 methanol : water (% v/v); (6) *F. johnsoniae* with DMSO+ *B. thailandensis* supernatant; and (7) *F. johnsoniae* with daidzein+ *B. thailandensis* supernatant. A volume of 1.04 µl was added to tubes containing daidzein (10 mg ml^−1^) or DMSO and a volume of 16.25 µl was added to tubes containing *B. thailandensis* supernatant or 50 : 50 methanol : water (% v/v). After additions of solvent or components, 200 µl aliquots from each tube was placed in a 96-well plate (Fig. S1, available in the online version of this article). The plate contained five independent replicates, two technical replicates/independent replicates for conditions 1–3 and three technical replicates/independent replicates for conditions 4–7 (90 samples). For the remaining six wells, 200 µl TSB_50_ was added to each well as a negative control. An initial OD_590_ reading was measured on a Tecan Infinite F500 Multimode Microplate Reader (Tecan Group Ltd, Männedorf, Switzerland). The plate was then incubated with shaking at 200 r.p.m. for 24 h at 27 °C. A final OD_590_ reading was taken after 24 h of incubation. A Wilcoxon rank sum test was used to compare all the controls to the *F. johnsonaie* culture control. All comparisons were insignificantly different (*q*-values>=0.82). For this reason, we only used the *F. johnsoniae* culture control (with no added solvents) to serve as a control for a follow-up Wilcoxon rank sum test with FDR correction that compared final OD_590_ across test conditions. Analyses were performed in R using the stats package [19]. Measurements were uploaded to R to generate boxplots using ggplot2 [20].

### Experimental evolution

**Fig. 1.**
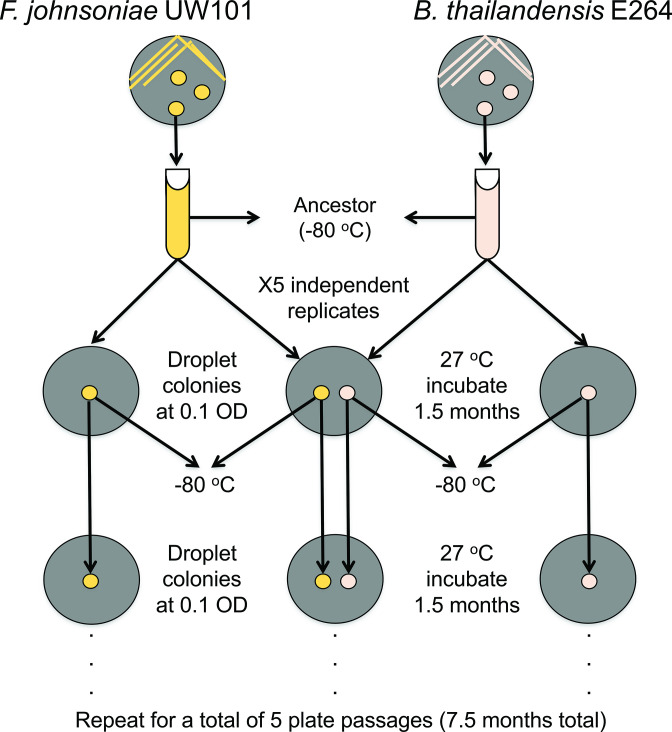
Schematic of (co)evolution experiment.

*B. thailandensis* E264 and *F. johnsoniae* UW101 were plated from freezer stocks onto TSA_50_. Plates were incubated for ~16 h at 27 °C. Single isolated colonies of *B. thailandensis* and *F. johnsoniae* were inoculated as separate cultures in 7 ml of TSB_50_ to serve as the ancestral cultures. Cultures were incubated with shaking at 200 r.p.m. for ~16 h at 27 °C. The following day, the cultures were pelleted by centrifugation at 5000 r.p.m. for 10 min at room temperature, the supernatant was removed and the cultures were resuspended in 1× phosphate-buffered saline (PBS). This process was repeated once more. The cultures were resuspended in PBS at a final volume of 5 ml. Ancestral freezer stocks were prepared by adding 750 ml of each overnight culture to 750 ml of 70 % glycerol (% v/v). All freezer stocks were stored at −80 °C. From the remaining cultures containing ancestral *B. thailandensis* and ancestral *F. johnsonaie*, the OD_590_ was measured on an Evolution 60S UV–visible spectrophotometer (Thermo Scientific) and each culture was diluted to an OD_590_ of 0.1 in PBS to prepare the evolution experiments. Ten microlitres of a culture (OD_590_ 0.1) was spotted onto M9 minimal salts agar plates containing 0.2 % glucose (M9 glucose; % v/v). Both strains were plated in isolation and co-plated (Fig. 1). When co-plated together, *B. thailandensis* and *F. johnsoniae* were spotted 14 mm apart. This allowed sufficient time for the strains to interact chemically without intergrowth of colonies. Five independent replicates were prepared, resulting in a total of 15 plates. The plates were wrapped with parafilm and incubated at 27 °C for 1.5 months.

After incubation, we performed a plate passage. Sterilized toothpicks were used for the collection of lawn growth. These cells were resuspended in 1 ml of PBS. For *B. thailandensis *, we preferentially collected radial colony growth that was growing toward *F. johnsoniae *. A section of radial colony growth was collected for *B. thailandensis* colonies in monocultures. The entirety of the *F. johnsoniae* was removed from each plate. First plate passage freezer stocks were prepared by adding 500 ml of each resuspended culture to 500 ml of 70 % glycerol (% v/v). From the remaining cultures, the OD_590_ was measured on an Evolution 60S UV–visible spectrophotometer (Thermo Scientific) and each culture was diluted to an OD_590_ of 0.1 in PBS. The plating scheme was repeated as previously described while preserving the previous replicate partnerships of the last coevolved experiment (e.g. *B. thailandensis* coevolved replicate 1 was replated with *F. johnsoniae* coevolved replicate 1). The plates were wrapped with parafilm and incubated at 27 °C for 1.5 months. This process was repeated for a total of five plate passages, resulting in a total experimentation time of 7.5 months.

### Measurements of radial colony growth of (co)evolved plates

Prior to a setting up another plate passage (after a 1.5 month incubation period), plates were imaged using an Epson Perfection V370 photo scanner. A ruler was placed in the scanner to scale pixels to millimetres for radial growth measurements. Images were uploaded to ImageJ2 for analysis [21, 22]. Radial growth was determined by measuring the distance from the centre of the colony to the furthest point of radial growth. Measurements were uploaded to R to generate boxplots using ggplot2 [20].

### Whole-genome sequencing

We generated whole genome sequencing data for mutational analysis from both *F. johnsoniae* and *B. thalandensis*. All replicates (monoculture and coevolution experiments) from the fifth plate passages and the ancestors were plated from freezer stocks onto TSA_50_ (11 freezer stocks in total). Plates were incubated for ~16 h at 27 °C. The following night, isolated colonies were inoculated into TSB_50_ medium and incubated with shaking at 200 r.p.m. for ~16 h at 27 °C. The following morning, DNA was extracted from all 11 cultures using the E.Z.N.A. Bacterial DNA kit (Omega Bio-tek, Norcross, GA, USA) according to the manufacturer’s instructions. DNA integrity was assessed from 260/280 and 260/230 ratios using a NanoDrop ND-1000 UV–visible spectrophotometer (Thermo Fisher Scientific, Waltham, MA, USA) and quantified using a Qubit 2.0 fluorometer (Invitrogen, Carlsbad, CA, USA). DNA samples were sent to the Microbial Genome Sequencing Center (MiGS, Pittsburgh, PA, USA) for whole-genome sequencing. Illumina DNA library preparations were performed at the MiGS facility according to standard operating protocols. Sequencing (2×151 bp) was performed on a NextSeq 2000 platform. A minimum of 200 Mbp of sequencing data was obtained with >Q30 reads. Low-quality bases were removed with Trimmomatic [23] using a sliding-window approach; reads were trimmed if the average Phred quality score was <20 in a 4 bp window. Reads were also removed if the read length was <70 bp. Average Phred quality scores of trimmed reads were assessed using FastQC [24]. Mutations were identified using *breseq* version 0.37.0 with default parameters [25] *B. thailandensis* E264 (accession numbers NC_007651 and NC_007650) and *F. johnsoniae* UW101 (accession number NC_009441) were used as reference sequences. The summary files from post-*breseq* analysis were used to determine total reads, percentage of reads mapped to reference sequences and average genome coverage. Sequencing statistics from whole-genome sequencing are provided in File S2. *Flavobacterium johnsoniae* UW101 and *Burkholderia thailandensis* E264 monoculture replicates, coevolved replicates, and ancestors whole genome raw sequence files from the coevolution are deposited in the NCBI Sequence Read Archive (BioProject ID PRJNA812898). Analysis code is available on GitHub at https://github.com/ShadeLab/Paper_Chodkowski_Coevolution_2022.

### TolC model prediction

The *F. johnsoniae* TolC protein sequence of interest (FJOH_RS06580) was downloaded from the National Center for Biotechnology Information (NCBI). The protein FASTA file was edited manually to remove 11 amino acids associated with the 33 bp deletion (amino acids 87–97). The protein FASTA file was also edited manually to create the nonsynonymous mutation (G83R). Then all three files were placed into SWISS-MODEL to model the protein structure of TolC [26]. The template used for rendering all models was SWISS-MODEL Template Library (SMTL) ID: 6wxi.1 [27]. The model from TolC wild-type (WT) was downloaded and uploaded into Swiss-PdbViewer to highlight amino acids associated with the 33 bp deletion [28]. The SWISS-MODEL homology target-template modelling reports are available at https://github.com/ShadeLab/Paper_Chodkowski_Coevolution_2022.

### Construction of mutants in *F. johnsoniae* ancestral strain

Single mutations of interest observed in the coevolved lines were engineered into the genome of the ancestral strain. These mutants were constructed following a previously described method [29], with the exception of an additional nested PCR step. All primers used in this study are listed in Table S3.

### Nested PCR

DNA extracted from coevolved lines for whole-genome sequencing was used as templates for PCR. Coevolved line 1 (CoE_Δ33_*tolC*) was used to amplify the 33 bp deletion in *tolC* (* F. johnsoniae* locus FJOH_RS06580; 261–293 in coding sequence), coevolved line 3 (CoE_G247A_*tolC*) was used to amplify the nonsynonymous mutation in *tolC* (* F. johnsoniae* locus FJOH_RS06580; G247A in coding sequence), and coevolved line 4 (CoE_792_793insG_*ragB*) was used to amplify the base insertion sequence in *ragB*/*susD* (* F. johnsoniae* locus FJOH_RS24865; 792_793insG in coding sequence). Nested PCR was performed to obtain enough DNA for downstream methods. For the first round of nested PCR, a 3.3 kbp fragment containing *tolC*Δ33 or *tolC*(G247A) was amplified using primers 1001 and 1002. A 3.5 kbp fragment containing *ragB*792_793insG was amplified using primers 1010 and 1011. The PCR reactions contained the reagents and volumes outlined in Table S4. The PCR conditions were as follows: 98 °C for 30 s, 98 °C for 10 s, 56 °C for 15 s and 72 °C for 70 s, repeated 29 times from step 2, followed by 72 °C for 10 min and hold at 4 °C. PCR products were run on a 0.8 % agarose (% w/v) gel at 100 V for 60 min. PCR bands at the correct fragment sizes were excised from the gel and extracted using Wizard SV Gel and PCR Clean-Up System (Promega Corporation, Madison, WI, USA). PCR products were quantified using a Qubit 2.0 fluorometer (Invitrogen, Carlsbad, CA, USA).

For the second round of PCR, a 3.2 kbp fragment containing *tolC*Δ33 or *tolC*(G247A) was amplified using primers 1003 (engineered XbaI site) and 1004 (engineered BamHI site). A 3.1 kbp fragment containing *ragB*792_793insG was amplified using primers 1012 (engineered XbaI site) and 1013 (engineered BamHI site). The PCR reactions contained the reagents and volumes outlined in Table S5. PCR conditions were as follows: 98 °C for 30 s, 98 °C for 10 s, 56 °C for 15 s and 72 °C for 70 s, repeated 29 times from step 2, followed by 72 °C for 10 min and hold at 4 °C. PCR products were run on a 0.8 % agarose (% w/v) gel at 100 V for 60 min. PCR bands at the correct fragment sizes were excised from the gel and extracted using Wizard SV Gel and PCR Clean-Up System (Promega Corporation, Madison WI, USA). PCR products were quantified using a Qubit 2.0 fluorometer (Invitrogen, Carlsbad, CA, USA).

### Plasmid isolation and purification

*Escherichia coli* DH5ɑmcr_pYT354 freezer stock was plated on lysogeny broth (LB) agar with ampicillin (100 µg ml^−1^) and incubated for ~16 h at 37 °C. A single colony was inoculated into 5 ml LB with ampicillin (100 µg ml^−1^) and incubated with shaking at 200 r.p.m. for ~16 h at 37 °C. Plasmid pYT354 was extracted *E. coli* DH5ɑmcr_pYT354 and purified the following morning using the E.Z.N.A. Plasmid DNA mini kit I Q-spin (Omega Bio-tek, Norcross, GA, USA) according to the manufacturer’s instructions.

### Restriction enzyme digestion and ligation

Separately prepared restriction enzyme double digestions were performed on purified PCR products from nested PCR round 2 and pYT354. The reaction reagents and volumes are outlined in Table S6. Reactions were incubated at 37 °C for 15 min. Reactions were then run on a 0.8 % agarose (% w/v) gel at 100 V for 60 min. Bands at the correct fragment sizes were excised from the gel and extracted using Wizard SV Gel and PCR Clean-Up System (Promega Corporation, Madison WI, USA). PCR products were quantified using a Qubit 2.0 fluorometer (Invitrogen, Carlsbad, CA, USA).

The PCR fragment containing *tolC*Δ33 was ligated into pYT354 to form pJC101, the PCR fragment containing *tolC* (G247A) was ligated into pYT354 to form pJC102, and the PCR fragment containing *ragB*792_793insG was ligated into pYT354 to form pJC103. Ligation reagents are outlined in Table S7. The reactions were incubated at room temperature for 10 min and then heat inactivated for 10 min at 65 °C.

### Preparation of heat shock competent cells

*E. coli* DH5ɑmcr freezer stock was plated on LB agar and incubated ~16 h at 37 °C. A single colony was inoculated into 5 ml LB and incubated with shaking at 200 r.p.m. for ~16 h at 37 °C. One millilitre of the overnight culture was diluted into 100 ml LB and incubated with shaking at 37 °C until the OD_590_ reached 0.3 (~ 2 h), as measured on an Evolution 60S UV–visible spectrophotometer (Thermo Scientific). The culture was placed on ice for 15 min. Two 45 ml aliquots were placed into two 50 ml falcon tubes and centrifuged at 4000 r.p.m. for 10 min at 4 °C. Supernatant was decanted, and the pellets were resuspended in 45 ml ice-cold 0.1 M CaCl_2._ The resuspended cultures were placed on ice for 30 min. Cultures were then centrifuged at 4000 r.p.m. for 10 min at 4 °C. Supernatant was decanted, and the pellets were resuspended in 4.5 ml ice-cold 0.1 M CaCl_2_ with 15 % glycerol (% v/v). The cultures were then distributed as 50 µl aliquots into microcentrifuge tubes. Competent cells were stored as freezer stocks at −80 °C until ready for use.

### Heat shock transformation

Heat shock competent cells (50 µl) were removed from the freezer and placed on ice for 20 min (four tubes, one for each ligation product and one pYT354 plasmid control). Two microlitres of ligation products (and 1 µl of 10 ng µl^−1^ pYT354 plasmid) were added to each tube and placed on ice for an additional 20 min. Cells were then heat shocked for 45 s at 42 °C. Heat-shocked cells were placed on ice for 2 min. One millilitre of super optimal broth (SOC) medium was added to each tube and the tubes were incubated with shaking at 200 r.p.m. at 37 °C for 1 h. Cells were pelleted by centrifugation at 4000 ***g*** for 2 min at 4 °C. The supernatant was removed (950 µl) and the remaining culture was plated on LB agar containing ampicillin (100 µg ml^−1^). Plates were incubated ~16 h at 37 °C. Successful transformants were inoculated into LB containing ampicillin (100 µg ml^−1^) and incubated ~16 h at 37 °C. Freezer stocks were made the following morning for triparental conjugation.

### Triparental conjugation and recombinant confirmation

pJC101, pJC103 and pJC104 in recombinant *E. coli* DH5αMCR needed to be transferred to the *F. johnsoniae* ancestral strain. This was introduced into *F. johnsoniae* by triparental conjugation as previously described [30] using recombinant *E. coli* DH5αMCR, *F. johnsoniae* ancestor and *E. coli* HB101 (carrying the helper plasmid pRK2013), except that the *sacB* and *ermF*-containing suicide vector was used to select for successful *F. johnsoniae* recombinants [29]. *F. johnsoniae* recombinants (reΔ33_*tolC*, reG247A_*tolC*, re792_793insG_*ragB*) were confirmed by Sanger sequencing at the Michigan State Genomics Core using primers 1005 and 1006 for tolCΔ33 or *tolC*(G247A) recombinants and primers 1014 and 1014 for the *ragB*792_793insG recombinant.

### Acetyl-CoA carboxylase carboxyl transferase subunit alpha (AccA) protein alignment

AccA sequences were downloaded from *F. johnsoniae* UW101 (protein ID: WP_011921560.1) and from *Salmonella enterica* serovar Typhimurium strain LT2 (protein ID: NP_459237.1) on NCBI and concatenated as a text file. The text file was uploaded to T-Coffee (version_11.00) for protein alignment using default parameters [31]. The FASTA alignment file from T-Coffee output was downloaded and used as input for BoxShade (version 3.21) using default parameters. The protein alignment was then uploaded to Inkscape for final edits.

### Extraction of *B. thailandensis* bioactive compound(s) and Kirby–Bauer assays

A freezer stock of *F. johnsoniae* was plated on TSA_50_ and grown for ~16 h at 27 °C. A loopful of lawn growth was transferred to 7 ml of M9 minimal salts 0.2% glucose (M9 glucose; % v/v) medium to achieve an initial OD_590_ of ~0.2 as measured on an Evolution 60S UV–visible spectrophotometer (Thermo Scientific). The culture was incubated with shaking at 200 r.p.m. for ~16 h at 27 °C. The next day, 5 ml of culture was transferred to 50 ml fresh M9 glucose medium in a 250 ml Erlenmeyer flask. The culture was incubated with shaking at 200 r.p.m. for 48 h at 27 °C. On the second day of the *F. johnsoniae* 48 h incubation, a freezer stock of *B. thailandensis* was plated on TSA_50_ and grown for ~16 h at 27 °C. A loopful of *B. thailandensis* lawn growth was transferred to 7 ml of M9 glucose medium to achieve an initial OD_590_ of ~0.2 as measured on an Evolution 60S UV–visible spectrophotometer (Thermo Scientific). The *B. thailandensis* culture was incubated with shaking at 200 r.p.m. for ~16 h at 27 °C.

At the completion of the *F. johnsoniae* 48 h incubation, the culture was transferred to a falcon tube and centrifuged at 5000 r.p.m. for 20 min at 4 °C. The supernatant was removed and filtered with a 0.22 µM PES filter, and ~50 ml was transferred to a 250 ml Erlenmeyer flask. Five millilitres of the overnight *B. thailandensis* culture was transferred to the 50 ml filtered spent *F. johnsoniae* M9 glucose medium. In addition, 250 µl 40 % glucose (% w/v) was added to the culture to reachieve ~0.2 % glucose (% v/v) in the M9 medium. The culture was incubated with shaking at 200 r.p.m. for 48 h at 27 °C. At the completion of the *B. thailandensis* 48 h incubation in the filtered spent *F. johnsoniae* medium, the culture was transferred to a falcon tube and centrifuged at 5000 r.p.m. for 20 min at 4 °C. The supernatant was removed, filtered with a 0.22 µM PES filter and transferred to a separatory funnel. Ten millilitres of DCM was added to the separatory funnel. The separatory funnel was agitated three times and the DCM layer was removed. The addition, agitation and removal of DCM was repeated two more times. The collected DCM layer was dried under nitrogen gas. The dried DCM extracts were reconstituted in 1 ml of a 50 : 50 methanol : water (% v/v) mixture. This mixture was used for the Kirby–Bauer assays.

Freezer stocks of the *F. johnsoniae* ancestor*,* co(evolved) strains from the fifth plate passage and recombinant strain, reΔ33_*tolC*, were plated on TSA_50_ and grown for ~16 h at 27 °C. A loopful of lawn growth was transferred and resuspended in 500 µl of 1× PBS. The cultures were pelleted by centrifugation at 5000 r.p.m. for 10 min at room temperature, the supernatant was removed and the cultures were resuspended in 500 µl 1× PBS. The OD_590_ of the cultures was measured on an Evolution 60S UV–visible spectrophotometer (Thermo Scientific). From the OD_590_ values, we calculated the volume of PBS to remove to achieve an OD_590_ of 10. The cultures were pelleted by centrifugation at 5000 r.p.m. for 10 min at room temperature, the calculated volumes of supernatant were removed and the cultures were resuspended in the remaining volume of 1× PBS. One hundred microlitres of each culture was placed on TSA_50_ using a cell spreader. TSA_50_ plates were used instead of M9 glucose agar plates because *F. johnsoniae* was incapable of growing overnight on this medium. Once dried, three 6 mm blank paper discs (BD BBL) we placed on each plate. Each disc received 20 µl of one of the following solutions: (1) *B. thailandensis* filtered spent medium grown in *F. johnsoniae* filtered spent medium (described above); (2) 50 : 50 methanol : water (% v/v) control; or (3) water control. Plates were incubated at ~16 h at 27 °C. Three independent replicates were prepared for each strain.

The following day, each plate was imaged using an Epson Perfection V370 photo scanner. A ruler was placed in the scanner to scale pixels to millimetres for zone of inhibition (ZOI) measurements. Images were uploaded to ImageJ2 for analysis [21, 22]. ZOIs were measured as the length between the outer perimeter of the disc to the point of observable growth. Measurements were uploaded to R to test for differences in ZOIs between strains using the Wilcoxon rank sum test. Plate images have been uploaded to GitHub: https://github.com/ShadeLab/Paper_Chodkowski_Coevolution_2022.

### Replating experiments

Strains of interest (co)evolved strains, ancestor strains, recombinants and transposon mutants were plated from freezer stocks onto TSA_50_. Plates were incubated for ~16 h at 27 °C. A loopful of lawn growth for each strain was inoculated as separate cultures in 7 ml TSB_50_. Cultures were incubated with shaking at 200 r.p.m. for ~16 h at 27 °C. The following day, the cultures were pelleted by centrifugation at 5000 r.p.m. for 10 min at room temperature, the supernatant was removed and the cultures were resuspended in 1× PBS. This process was repeated once more. The cultures were resuspended in PBS at a final volume of 5 ml. The OD_590_ was measured on an Evolution 60S UV–visible spectrophotometer (Thermo Scientific) and each culture was diluted to an OD_590_ of 0.1 in PBS. Ten microlitres of a culture (OD_590_ 0.1) was spotted onto M9 minimal salts agar plates containing 0.2 % glucose (M9 glucose; % v/v). Strains were either plated in isolation or co-plated together. When co-plated together, strains were spotted 14 mm apart. Plates were incubated at 27 °C for various amounts of time. *B. thailandensis* transposon mutants were acquired from the Manoil laboratory [32].

## Results

### *F. johnsoniae* is inhibited by bioactive compound(s) produced by *B. thailandensis*

**Fig. 2.**
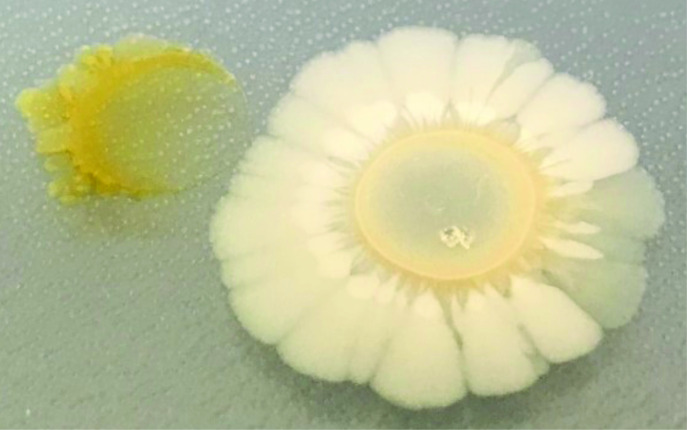
A bioactive compound is produced by *B. thailandensis* that inhibits *F. johnsoniae *. *B. thailandensis* (right) and *F. johnsoniae* (left) were co-plated on M9-glucose agar at a distance that allowed for chemical interactions. An unidentified antibiotic(s) inhibited *F. johnsoniae *.

We first observed that *B. thailandensis* inhibited *F. johnsoniae* when co-plated on M9 agar plates (Fig. 2). *B. thailandensis* exhibited radial growth on all edges along the circumference of the colony, while the *F. johnsoniae* colony proximal to *B. thailandensis* was inhibited. However, the distal end of the *F. johnsoniae* colony grew away from *B. thailandensis *, suggesting that *F. johnsoniae* may have intrinsic mechanisms to reduce antibiotic susceptibility. We note that, despite efforts to do so, we were unable to purify and directly link the antibiotic(s) that inhibited *F. johnsoniae *. We still use the terminology antibiotic(s) to refer to any number of compounds that may be inhibiting *F. johnsoniae *, whether that was an antibiotic derived from a BSGC or a by-product of *B. thailandensis* metabolism that was bioactive against *F. johnsoniae *.

### Efflux allows *F. johnsoniae* to grow in the presence of a *B. thailandensis *-produced antibiotic

**Fig. 3.**
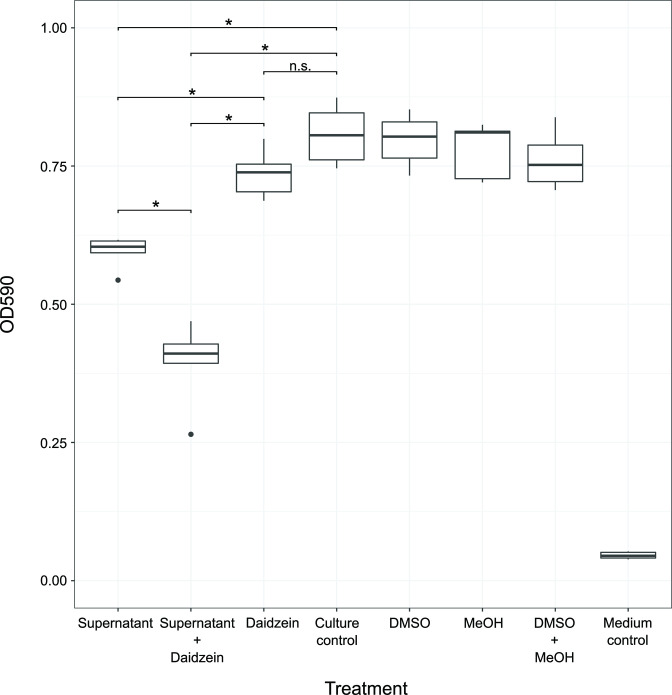
*
F. johnsoniae* efflux system contributes to the extrusion of a *B. thailandensis *-produced antibiotic(s). An end point growth measurement (n=5) was taken after *F. johnsoniae* 24 h incubation with either *B. thailandensis* culture supernatant (Supernatant), with the efflux pump inhibitor (Daidzein), or with a combination of the supernatant and daidzein. An untreated *F. johnsoniae* culture (Culture control), a *F. johnsoniae* culture with dimethyl sulfoxide (DMSO), a *F. johnsoniae* culture with methanol (MeOH), a *F. johnsoniae* culture with both solvents (DMSO + MeOH), and blank TSB50 medium served as controls. The bottom and top of each boxplot are the first and third quartiles, respectively, and the line inside the box is the median. The whiskers extend from their respective hinges to the largest value (top), and smallest value (bottom) was no further away than 1.5 times the interquartile range. Black points on a boxplot represent outliers that were greater than 1.5 times the interquartile range. A Wilcoxon Rank-Sum test was performed comparing all treatments to the culture control. * q-value <0.05, n.s.: not significant.

Given the growth pattern of *F. johnsoniae* when co-plated with *B. thailandensis *, we hypothesized that efflux contributed to *F. johnsoniae* as an intrinsic mechanism to reduce antibiotic susceptibility. We collected the organic fraction from spent supernatant of *B. thailandensis* grown in monoculture, which contained the antibiotic(s). We then treated *F. johnsoniae* cultures with the supernatant alone and in combination with daidzein, an efflux pump inhibitor [33]. While the supernatant inhibited *F. johnsoniae *, daidzein did not, and the supernatant with daidzein significantly inhibited *F. johnsoniae* more than the supernatant alone (Figs 3 and S1). This suggested that *F. johnsoniae* had intrinsic mechanisms to reduce antibiotic susceptibility via efflux and extrusion and that these intrinsic mechanisms of antibiotic tolerance could allow for mutational acquisition to further decrease antibiotic susceptibility. We tested this hypothesis by performing a coevolution experiment.

### Coevolutionary outcomes of *B. thailandensis *–* F. johnsoniae* interactions

**Fig. 4.**
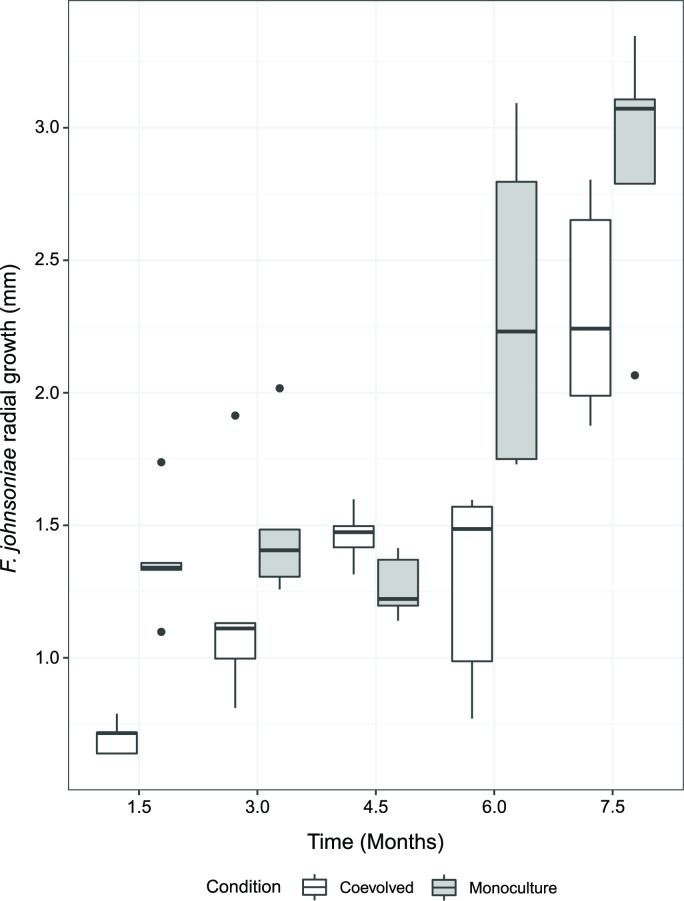
*
F. johnsoniae* trends toward increased growth success with each plate passage in the presence of *B. thailandensis *. Images were taken at the end of each plate passage (1.5 mos) to quantify radial growth for both *F. johnsoniae* monoculture evolved (gray bars) lines and *F. johnsoniae* coevolved (white bars) lines (n=5 independent replicated per time point). Radial colony growth was measured from the center of the colony to the point of furthest growth on the agar plate. The bottom and top of each box are the first and third quartiles, respectively, and the line inside the box is the median. The whiskers extend from their respective hinges to the largest value (top), and smallest value (bottom) no further away than 1.5× the interquartile range. Black points on a boxplot represent outliers that were greater than 1.5 times the interquartile range.

To better understand the underlying factors contributing to *F. johnsoniae* antibiotic susceptibility, we next asked how the susceptibility to antibiotic(s) in *F. johnsoniae* would change when coevolved with *B. thailandensis *. We performed an agar-based coevolution experiment with 10 µl volume of two liquid overnight cultures spotted 14 mm apart to allow for extracellular chemical interactions. These cultures were allowed to grow into colonies and passaged onto another plate before intergrowth of the colonies could occur (Fig. 1). The colonies spotted were either one of each of *B. thailandensis* and *F. johnsoniae* (‘coevolved’), or either *B. thailandensis* or *F. johnsoniae* in isolation (‘monoculture’). All monoculture controls were grown in parallel to the coevolved lines (Fig. 1). We observed that the radial growth increased between the fifth plate passage and the first plate passage within both the monoculture evolved and coevolved lines (Wilcoxon rank sum; *P*-values <0.01), suggesting increased growth capabilities on the M9 glucose medium over time. The radial growth of *F. johnsoniae* generally increased with each successive plate passage (Figs 4 and S2). In addition, *B. thailandensis* substantially inhibited *F. johnsoniae* on the first plate (Fig. S2), as the radial growth of the coevolved lines was significantly less than the radial growth of the monoculture lines (*q*-value=0.039). In successive plate passages, the radial growth in coevolved lines was insignificantly different to that in the monoculture evolved lines (*q*-values>=0.15), with the exception of plate passage 4 (*q*-value=0.039). This suggested that coevolved lines of *F. johnsoniae* were becoming less susceptible to *B. thailandensis *-produced antibiotics. Indeed, when a freezer stock from a coevolved *F. johnsoniae* line and a freezer stock from the corresponding monoculture *F. johnsoniae* line were plated with the *B. thailandensis* ancestor, *F. johnsoniae* from the coevolved line displayed more radial growth and thus less antibiotic susceptibility as compared to the evolved monoculture control (Fig. S3). Interestingly, colonies from the coevolved *F. johnsoniae* lines were also able to resist colony invasion by *B. thailandensis,* while colonies from the evolved monoculture lines could not (Fig. S4). Overall, these results suggest that coevolved *F. johnsoniae* evolved decreased susceptibility to antibiotics as an outcome of long-term exposure to and engagement with *B. thailandensis *.

### Whole-genome sequencing reveals genetic signatures of reduced antibiotic susceptibility

We performed whole-genome sequencing to discover mutations that may have contributed to reduced antibiotic susceptibility in the *F. johnsoniae* coevolved lines. We sequenced a clonal isolate from the *F. johnsoniae* ancestor and clonal isolates from each of the five independent coevolved and monoculture evolved lines from the final (fifth) plate passage. We detected mutations in all coevolved and monoculture-evolved isolate genomes compared to the ancestor (Table 1, File S1). *F. johnsoniae* lines coevolved with *B. thailandensis* acquired mutations that were distinct from acquired mutations in *F. johnsoniae* lines grown in monocultures (Table 2, Fig. S5).

**Table 1.** Summary of mutation types observed in *F. johnsoniae* from the (co)evolution experiment. Mutations are from five representative clonal isolates after the final (fifth) plate passage and the ancestor

* F. johnsoniae* isolate	Total	Insertion	Deletion	Nonsynonymous	Synonymous	Nonsense
Coevolved rep 1	6	2	1	3	0	0
Coevolved rep 2	5	0	2	3	0	0
Coevolved rep 3	6	0	0	5	1	0
Coevolved rep 4	5	1	1	3	0	0
Coevolved rep 5	5	1	2	2	0	0
Evolved monoculture rep 1	6	1	3	2	0	0
Evolved monoculture rep 2	7	1	3	2	0	1
Evolved monoculture rep 3	6	0	4	2	0	0
Evolved monoculture rep 4	6	2	1	3	0	0
Evolved monoculture rep 5	3	0	2	0	0	1
Ancestor*	2	0	1	1	0	0

*Ancestor mutations that were also observed in evolved line(s) were not included in the tally of evolved line mutations.

**Table 2.** Distinctions and overlaps of loci with mutations unique to the *F. johnsoniae* lines that were coevolved with *B. thailandensis *. These mutations were detected in at least one coevolved line and not present in any of the evolved *F. johnsoniae* monocultures

* F. johnsoniae * *locus*	Annotation	Rep 1	Rep 2	Rep 3	Rep 4	Rep 5
FJOH_RS00255	Acetyl-CoA carboxylase carboxyltransferase subunit alpha	–	–	C479A	–	–
FJOH_RS02320	Aminomethyl-transferring glycine dehydrogenase	–	–	–	–	Δ1 bp (504)
FJOH_RS04780	Hypothetical protein	G329C	–	–	–	–
FJOH_RS06580	TolC family protein	Δ33 bp (261–293)	Δ33 bp (261–293)	G247A	–	Δ33 bp (261–293)
FJOH_RS07830	NAD(P)/FAD-dependent oxidoreductase	–	C148A	–	–	–
FJOH_RS09515	LyTR family DNA-binding domain-containing protein	–	G46A	–	–	C44T
FJOH_RS09520	Histidine kinase	825insG	–	G496A	Δ1 bp (621)	–
FJOH_RS11170	Gfo/Idh/MocA family oxidoreductase	–	–	G273C	–	–
FJOH_RS12240	AIR synthase-related protein	–	–	C791T	–	–
FJOH_RS14175	Response regulator transcription factor	–	C269T	–	–	–
FJOH_RS20510	TetR/AcrR family transcriptional regulator	201insT	–	–	–	–
FJOH_RS21875	Glycoside hydrolase	T1489C	–	–	–	–
FJOH_RS24865	RagB/SusD family nutrient uptake outer membrane protein	–	–	–	793insG	–
FJOH_RS25290	Phosphoribosylanthranilate isomerase	–	–	–	T50C	–

**Fig. 5.**
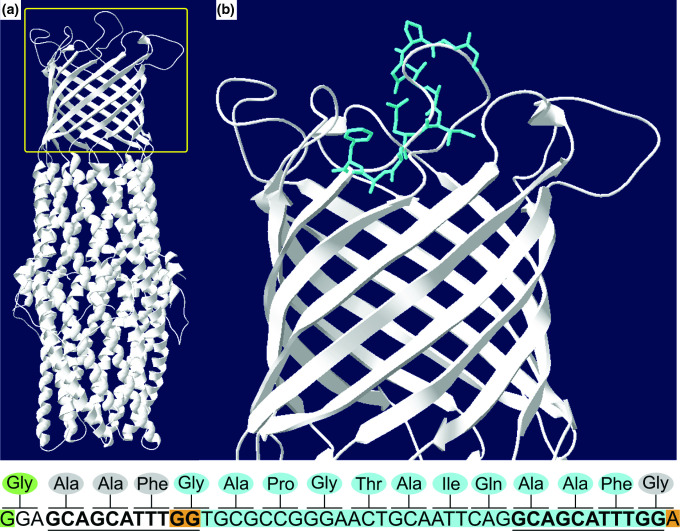
A *tolC* 33 bp deletion is located on a TolC extracellular loop. The TolC protein (A) contains a α-helical trans periplasmic tunnel, a β-barrel channel embedded in the outer membrane, and extracellular loops at the cell surface. The yellow box represents the inset in (B), where the 11 amino acids corresponding to the 33 bp deletion are highlighted in blue. These amino acids are part of one of the extracellular loops. The nucleotide sequence is shown below the image, representing bps 247-294 (amino acids 83-98) in the FJOH_RS06580 coding sequence. The corresponding amino acids are shown above each codon. Nucleotides highlighted in blue represent the 33 bp deletion. Nucleotides highlighted in orange show how the deletion was in-frame. The 11 bp direct repeats are in bold and underlined. The nucleotide highlighted in green is associated with the nonsynonymous mutation (G247A in the coding sequence, G83R in TolC). TolC from *E. coli* was used as the template (SMTL ID: 6wxi.1) to construct the target *F. johnsoniae* WT TolC model (SWISS-MODEL: GMQE= 0.6, Seq ID=19.06) shown in the figure. The *F. johnsoniae* TolC model generated from removal of 33 bp still maintains equivalent modeling quality (SWISS883 MODEL: GMQE=0.61, Seq ID=19.06).

*F. johnsoniae* evolved isolates harboured mutations in an efflux outer-membrane protein, TolC, and could be evidence of an acquired mutation that would reduce antibiotic susceptibility (Table 2). A *tolC* mutation was observed in four out of the five coevolved lines. While *F. johnsoniae* contains 16 *tolC* genes (Table S1), the same gene was mutated in 4 independent replicates. However, the TolC harbouring mutations in coevolved isolates (FJOH_RS06580) only had 24.2 % or less protein sequence identity with all other TolC proteins in *F. johnsoniae* (Table S2). Furthermore, there was evidence of parallel evolution at the nucleotide level, as 3/5 of the coevolved lines had the same *tolC* 33 bp deletion at the same *tolC* locus (File S1). This deletion was from nucleotides 261–293 in the FJOH_RS06580 coding sequence, resulting in an in-frame deletion (Fig. 5). This deletion affected one of the extracellular loops of TolC. The WT sequence revealed a 11 bp direct repeat, occurring before the deletion and representing the last 11 bp of the 33 bp deletion. This suggests that the deletion may have occurred during a replication deletion event. The other mutation in FJOH_RS06580 (coevolved line 3) was a single-nucleotide polymorphism (G247A in the coding sequence) that resulted in a nonsynonymous mutation (G83R). Protein modelling suggested that this mutation resulted in a decreased diameter of the efflux channel (Fig. S6). Coevolved line 4 did not harbour a mutation in FJOH_RS06580 but instead had a unique bp insertion in a *ragB*/*susD* nutrient uptake outer-membrane protein (FJOH_RS24865). Guanine was inserted between nucleotide positions 792 and 793 in the coding sequence for FJOH_RS24865. This frameshift mutation resulted in a premature termination codon 6 bp downstream of the insertion, likely rendering the protein nonfunctional. Mutations were also uniquely found in regulators of coevolved isolates, including a small insertion in a TetR/AcrR family transcriptional regulator in coevolved isolate 1, a nonsynonymous mutation in an OmpR family response regulator in coevolved isolate 2, and a nonsynonymous mutation in a LytTR family response regulator within the same loci of coevolved isolates 2 and 5. These regulators may have indirect effects on antibiotic susceptibility (e.g. up-/downregulation of efflux pumps).

Mutations were also observed in (co)evolved *B. thailandensis* lines (File S1). Most mutations were unique to individually sequenced isolates. Exact mutational parallelism was present, (e.g. flagellar assembly protein FilH, TetR/AcrR transcriptional regulator and efflux periplasmic adaptor subunit) but was observed across both monoculture evolved lines and coevolved lines, indicating that these mutations were not acquired due to the presence of *F. johnsoniae *.

One mutation of interest was observed in *B. thailandensis* coevolved line 4: a 7 bp insertion in BTH_II1238. This gene codes for *btaP*, a metallo-β-lactamase involved in the production of the bactobolin antibiotic [34]. The insertion resulted in a premature stop codon, rendering the protein non-functional. A previous study showed abrogated bactobolin production in a *btaP* knockout [35], meaning that the insertion observed in our study likely abrogated bactobolin production as well. To ascertain that *F. johnsoniae* inhibition persists in the presence of *B *. *thailandensis* with abrogated bactobolin production, we co-plated *F. johnsoniae* with a *B. thailandensis btaK*::T23 transposon mutant. We had possession of this transposon mutant, and, similar to the *btaP* mutant, the *btaK* (BTH_II1233) mutant has been confirmed to abrogate bactobolin production [34]. As expected, we found that the *B. thailandensis btaK*::T23 transposon mutant inhibited *F. johnsoniae* despite the inability to produce bactobolin (Fig. S7), suggesting either that bactobolin was ineffective against *F. johnsoniae* or that multiple antibiotics were involved in *F. johnsoniae* inhibition. Overall, there was no mutational genomic evidence to help to inform the *B. thailandensis *-produced antibiotic(s) that inhibits *F. johnsoniae *.

### The FJOH_RS06580 *tolC* 33 bp deletion reduces antibiotic susceptibility

**Fig. 6.**
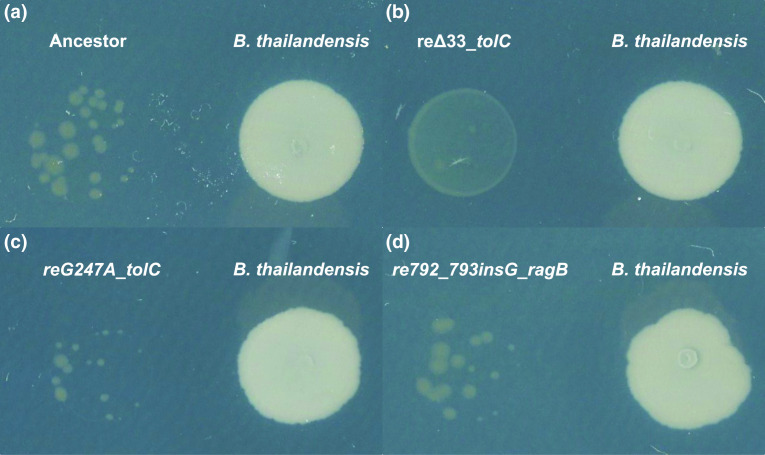
The *tolC* 33 bp deletion reduces antibiotic susceptibility. The ancestor (A) and recombinant strains of *F. johnsoniae* ancestor (C-D) were co-plated with the *B *. *thailandensis* ancestor. The recombinant strain with the *tolC* 33 bp deletion (B) is the least inhibited by *B. thailandensis* compared to the *F. johnsoniae* ancestor and other *F. johnsoniae* recombinant strains. Plates were imaged after a week of incubation.

We asked whether the mutations in *tolC* or the mutation in *ragB*/*susD* observed in coevolved *F. johnsoniae* lines would reduce antibiotic susceptibility to the *B. thailandensis *-produced antibiotic(s). These mutations were amplified from the coevolved *F. johnsoniae* lines and recombined into the ancestor so that the ancestor would only harbour one of these mutations and not any of the additional mutations observed in the coevolved lines. The strains and plasmids used to make recombinant *F. johnsoniae* are outlined in Table 3. Ancestor *B. thailandensis* inhibited the *F. johnsoniae* ancestor and recombinant strains reG247A_tolC and re792_793insG_ragB (Fig. 6). In fact, reG247A_tolC appeared more inhibited compared to the *F. johnsoniae* ancestor, suggesting that a decreased diameter to the TolC efflux channel (inferred from protein structure predicted by SWISS-MODEL) is detrimental to fitness. In contrast, recombinant strain reΔ33_tolC was the least inhibited when plated with ancestor *B. thailandensis *, suggesting that the 33 bp deletion reduces susceptibility to an antibiotic(s) (Fig. 6b). Thus, it appears that *F. johnsoniae* uses intrinsic mechanisms to reduce antibiotic susceptibility (e.g. efflux pumps, Fig. 3) but also acquired a mutation in the outer-membrane protein of an efflux system that further reduced susceptibility to a *B. thailandensis *-produced antibiotic(s). The acquired mutation evolved independently in 3/5 of the coevolved isolates at the same *tolC* locus. However, the 33 bp deletion in *tolC* alone did not reduce antibiotic susceptibility to the same degree as observed in the coevolved lines, as the coevolved lines harbouring the *tolC* 33 bp deletion with additional mutations were less susceptible to *B. thailandensis *-produced antibiotic(s) compared to the recombinant strain reΔ33_tolC (Fig. S8). This suggests that coevolved lines harbouring the *tolC* 33 bp mutation may contain synergistic mutations contributing to further reductions in antibiotic susceptibility.

**Table 3.** Strains and plasmids used in this study

Strain or plasmid	Description	Source or reference
* Escherichia coli* strains		
DH5αMCR	Strain used for general cloning	Life Technologies (Grand Island, NY, USA)
HB101	Strain used with pRK2013 for triparental conjugation	[63, 64]
DH5αMCR_pYT354	pYT354 in DH5amcr; *sacB*-containing suicide vector	[29]
* B. thailandensis* strain		
E264 (ATCC 700388)	Wild-type	[65]
* F. johnsoniae* strains		
UW101 (ATCC 17061)	Wild-type	[66]
CoE_Δ33_*tolC*	Coevolved strain containing a 33 bp (261–293) deletion in *tolC* (FJOH_RS06580)	This study
CoE_G247A_*tolC*	Coevolved strain containing a nonsynonymous mutation (G247A) in *tolC* (FJOH_RS06580)	This study
CoE_792_793insG_*ragB*	Coevolved strain containing an insertion (G) between bp 792 and 793 in *ragB*/*susD* (FJOH_RS24865)	This study
reΔ33_*tolC*	*tolC* (FJOH_RS06580) 33 bp deletion placed in ancestor	This study
reG247A_*tolC*	*tolC* (FJOH_RS06580) G247A mutation placed in ancestor	This study
re792_793insG_*ragB*	*ragB*/*susD* (FJOH_RS24865) bp (G) insertion placed between bp 792 and 793 in ancestor	This study
Plasmids		
pYT354	*sacB*-containing suicide vector; Ap^r^ (Em^r^). pYT354 is modified from pYT313 with different multiple cloning sites	[29]
pJC101	Construct used to replace ancestral native *tolC* (FJOH_RS06580) with *tolC* 33 bp deletion	This study
pJC102	Construct used to replace ancestral native *tolC* (FJOH_RS06580) with *tolC* G247A	This study
pJC103	Construct used to replace ancestral native *ragB*/*susD* (FJOH_RS24865) with *ragB*/*susD* 792_793insG	This study
pRK2013	Helper plasmid for triparental conjugation; Km^r^	[64]

Ap^r^, ampicillin resistance, 100 μg ml^−1^; Km^r^, kanamycin resistance, 30 μg ml^−1^; Em^r^, erythromycin resistance, 100 μg ml^−1^ for *F. johnsoniae*. Antibiotics resistance phenotypes listed in parentheses (Em) are those expressed in *F. johnsoniae* but not in *E. coli*. Antibiotics resistance phenotypes listed not in parentheses (Ap, Km) are those expressed in *E. coli* but not in *F. johnsoniae*.

We then quantified the extent of *F. johnsonaie* antibiotic susceptibility. We grew *B. thailandensis* in spent *F. johnsoniae* medium and extracted the organic fraction from the spent *B. thailandensis* medium. The organic layer contained unknown compound(s) that were bioactive against *F. johnsoniae *. A Kirby–Bauer disc diffusion assay was performed to quantify the ZOI of our *F. johnsoniae* strains (Table 4). The ZOIs in coevolved strains were significantly smaller than the ZOIs in monoculture evolved strains (Wilcoxon rank sum; *P*-value <0.001). However, the ZOIs in coevolved replicates were not significantly different from the ZOI in the ancestor (Wilcoxon rank sum; *q*-values>=0.6). In addition, the ZOI in the recombinant strain, reΔ33_*tolC*, did not significantly differ from the ZOI in the ancestor (Wilcoxon rank sum; *q*-value=1.0). Despite this observation, all coevolved strains and the reΔ33_*tolC* recombinant strain grew better than the ancestor when plated together on the same plate with *B. thailandensis* (Fig. S9). Coevolved strains, but not the recombinant strain, grew better than the monoculture evolved strains, consistent with the Kirby–Bauer results. Taken together, these data suggest that coevolved strains are less susceptible to antibiotic(s) produced by *B. thailandensis *.

**Table 4.** Results of antimicrobial activity observed in spent *B. thailandensis* medium against *F. johnsoniae* strains represented by zone of inhibition (ZOI)

Strain	ZOI (mm)		
Ancestor	2.62	2.63	3.10
Evolved monoculture rep 1	5.50	5.39	6.20
Evolved monoculture rep 2	4.90	5.55	4.61
Evolved monoculture rep 3	5.90	5.53	3.25
Evolved monoculture rep 4	3.96	4.72	4.75
Evolved monoculture rep 5	5.41	5.17	5.14
Coevolved rep 1	1.31	2.14	1.33
Coevolved rep 2	2.00	2.00	2.17
Coevolved rep 3	0	0	0
Coevolved rep 4	2.06	2.04	2.07
Coevolved rep 5	1.97	3.08	2.31
reΔ33_*tolC*	2.48	3.17	3.11

### Thailandamide is one of the bioactive molecules that inhibits *F. johnsoniae*

**Fig. 7.**
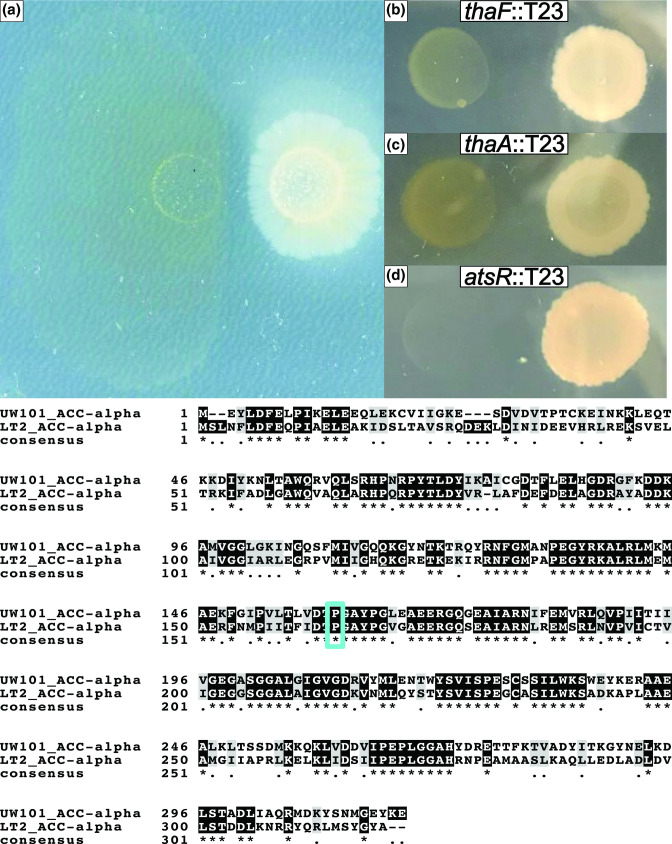
Thailandamide is bioactive against *F. johnsoniae *. While the nonsynonymous mutation in *tolC* did not reduce antibiotic susceptibility (present in *F. johnsoniae* coevolved line 3), *F. johnsoniae* coevolved line 3 still displayed a reduction in antibiotic susceptibility at the end of plate passage five (A). *B. thailandensis* transposon mutants in *thaF* (B) and thaA (C) have decreased inhibition toward *F. johnsoniae* while an *atsR* transposon mutant has increased inhibition of *F. johnsoniae* (D). Below the panels is an amino acid sequence alignment between the *F *. *johnsoniae* AccA and S. enterica AccA. The asterisks indicate positions within the proteins that were identical. The blue box highlights the alignment of P160 in *F. johnsoniae* and P164 in *S. enterica *.

The nonsynonymous mutation in *F. johnsoniae* coevolved line 3 *tolC* did not reduce susceptibility to the *B. thailandensis *-produced antibiotic(s). However, *F. johnsoniae* coevolved line 3 displayed reduced antibiotic susceptibility despite harbouring this mutation (Fig. 7a). This *F. johnsoniae* coevolved line 3 also contained a unique nonsynonymous mutation in acetyl-CoA carboxylase carboxyltransferase subunit alpha (*accA*; Table 2). This was a C479A nonsynonymous mutation in the coding sequence of *accA* that resulted in a P160Q alteration in AccA. This mutation was similar to a P164Q mutation in AccA from *S. enterica* serovar Typhimurium strain LT2 that conferred resistance to thailandamide from *B. thailandensis* [36]. The P164Q mutation from *S. enterica* aligned with the P160Q mutation observed in our coevolved line (Fig. 7). Thus, we hypothesized that thailandamide was one of the antibiotics responsible for inhibition of *F. johnsoniae *.

We plated the *F. johnsoniae* ancestor with *B. thailandensis thaF* (BTH_II1675), *thaA* (BTH_II1681) and *atsR* (BTH_I0633) transposon mutants. *thaF* encodes a polyketide synthetase trans-AT domain directly involved in the biosynthesis of thailandamide, *thaA* encodes a LuxR-type regulator in the thailandamide biosynthetic gene cluster and *astR* encodes a global regulator. ThaA positively regulates the thailandamide biosynthetic gene cluster, while AtsR negatively regulates the thailandamide biosynthetic gene cluster. We found that *thaF*::T23 and *thaA*::T23 mutants decreased inhibition of *F. johnsoniae* (Fig. 7b, c), while the *atsR*::T23 mutant increased inhibition of *F. johnsoniae* (Fig. 7d). This suggests that thailandamide is bioactive against *F. johnsoniae *. However, *F. johnsoniae* was still slightly inhibited when plated with *thaF*::T23 and *thaA*::T23 mutants, which also suggests that in addition to thailandamide, there may be another *B. thailandensis *-produced antibiotic that is inhibiting *F. johnsoniae *. However, we were unable to determine this molecule.

## Discussion

We performed an experimental coevolution study between a strain capable of antibiotic production (* B. thailandensis *) and an antibiotic-susceptible strain (* F. johnsoniae *). Our findings show how long-term interspecies interactions facilitated through chemical exchange via diffusion in agar can result in the acquisition of mutations that reduce antibiotic susceptibility. Analysis of mutations of *F. johnsoniae* coevolved lines as compared to lines evolved in monoculture revealed that reduced antibiotic susceptibility was achieved via a mutation in an efflux outer-membrane protein. Specifically, a 33 bp deletion in *tolC*, which eliminated 11 amino acids that were part of an extracellular loop in TolC, reduced antibiotic susceptibility in coevolved *F. johnsoniae* lines. Parallel evolution was observed for this mutation, as 3/5 coevolved isolates harboured the same 33 bp deletion within the same *tolC* locus. Decreased antibiotic susceptibility was also achieved through a nonsynonymous mutation in *accA*. While the *accA* mutation was not directly assessed for antibiotic susceptibility in our study, the same mutation was spontaneously derived in a *Salmonella* strain that conferred resistance to *B. thailandensis *-derived thailandamide [36]. This led us to hypothesize that thailandamide was the antibiotic inhibiting *F. johnsoniae. B. thailandensis* transposon mutants with abrogated thailandamide production confirmed that thailandamide was bioactive against *F. johnsoniae *, but slight inhibition was observed, again suggesting that multiple antibiotics are inhibiting *F. johnsoniae *.

Performing the experimental coevolution on agar plates created a heterogenous environment that facilitated the evolution of decreased antibiotic susceptibility. *F. johnsoniae* growth at the distal end of the colony was permitted because of an antibiotic concentration gradient that was established via diffusion. Low-dose antibiotics likely upregulated intrinsic mechanisms of resistance (e.g. efflux pumps) that conferred low levels of resistance [37]. The ability to survive exposure to antibiotics via intrinsic mechanisms [38, 39] can provide the opportunity for mutational acquisition of resistance [13, 14]. This was demonstrated experimentally in a seminal study that found that a heterogenous environment increased the rate of adaptation to antibiotics with as few as 100 bacteria in the initial inoculum [40]. This approach has been expanded to show how the initial adaptation to low levels of antibiotics facilitates adaptations to high levels of resistance [41]. Thus, evolutionary adaptations to antibiotic resistance can be fostered in heterogenous environments that would otherwise not be achieved in a uniform environment [42, 43].

Reduced susceptibility to the *B. thailandensis* antibiotic(s) was observed in *F. johnsoniae* coevolved lines that had a 33 bp deletion in *tolC*. The occurrence of the 11 bp directed repeats may support a replication misalignment event that led to the 33 bp deletion [44, 45]. TolC forms the outer-membrane channel part of resistance-nodulation-division (RND) efflux transporters [46, 47]. *tolC* (FJOH_RS06580) is located in an operon that includes the remaining components necessary for a functional efflux pump. This includes a TetR/AcrR family transcriptional regulator (FJOH_RS06575), an efflux transporter periplasmic adaptor subunit (FJOH_RS06585) and a multidrug efflux pump permease subunit (FJOH_RS0690). We note that we were unable to confirm the RND-type transport system of this operon. Elimination of 11 amino acids from an extracellular loop in TolC may result in TolC more frequently adopting an open conformation, which could increase the rate of antibiotic extrusion. ‘Leaky’ TolC mutants have been characterized, but these mutations occurred at the periplasmic end of TolC [48, 49]. In fact, mutational studies of the TolC extracellular loops appear uncommon but may present a novel mechanism for antibiotic resistance [50]. One study carried out a mutational analysis in OprM, a TolC homologue. Two separate insertion mutants were created in external loops of the OprM β-barrel. Modest reductions in channel conductance were observed for these mutants, but this was correlated with either unchanged or reduced MICs to an array of antimicrobials. Regardless, the authors demonstrated that external loops contribute to the control of passage of certain substrates [51].

The *F. johnsoniae* recombinant strain harbouring the 33 bp deletion in *tolC* had reduced antibiotic susceptibility, but not to the same degree as the *F. johnsoniae* coevolved lines to the *B. thailandensis *-produced antibiotic(s). Additional mutations in the coevolved lines are likely to provide further reductions in antibiotic susceptibility. For example, coevolved line 1 also harboured a 1 bp insertion (T) at position 4 734 734 in FJOH_RS20510, annotated as a TetR/AcrR family transcriptional regulator. This results in a nonsense mutation that would render the protein nonfunctional. TetR regulators are typically negative regulators, so a nonfunctional TetR regulator would lead to increased expression of efflux pump systems [52]. We note that this occurred in FJOH_RS20510 and not FJOH_RS06575, but some TetR regulators have multiple targets and TetR from FJOH_RS20510 could also be negatively regulating the FJOH_RS06580-FJOH_RS06585-FJOH_RS06590 efflux system [53]. The remaining coevolved lines with the 33 bp deletion in *tolC* (line 2 and line 5) also had mutations in transcriptional regulators (OmpR and LytTR). Mutations in OmpR may have a potential link to antibiotic resistance by altering expression of the major porin, OmpF, or other genes controlled under the EnvZ/OmpR two-component regulatory system [54]. The potential contributions of mutations in LytTR to reduced antibiotic susceptibility is unknown.

While it was clear that differences in antibiotic susceptibility emerged between the monoculture and coevolved lines, there were both congruencies and discrepancies between Kirby–Bauer assay results (Table 4) and qualitative plate assays (Fig. S9). To start, the decreased antibiotic susceptibility in coevolved strains was consistent between both assays. Quantitatively, the coevolved strains had a modest decrease in ZOIs compared to the ancestor and a significant decrease compared to monoculture evolved strains. Qualitatively, the coevolved strains were the least susceptible to the *B. thailandensis *-produced antibiotic(s). The monoculture evolved strains had a modest increase in ZOIs compared to the ancestor despite qualitatively being less susceptible to the *B. thailandensis* antibiotic(s). Lastly, the ZOI of the recombinant strain, reΔ33_*tolC*, was not different from the ancestor but it was qualitatively less susceptible to the *B. thailandensis* antibiotic(s). These differences in quantitative versus qualitative outcomes could have arisen for a few reasons. The Kirby–Bauer assays were performed from antibiotic(s) concentrated from spent liquid culture. The antibiotic profile of *B. thailandensis* in liquid culture could be different from the profile on agar medium. Similarly, the spent supernatant from the Kirby–Bauer assay was obtained from strains that did not directly interact with one another. There could be a dynamic chemical interaction between *F. johnsonaie* and *B. thailandensis* when co-plated that leads to a unique antibiotic profile not achieved by growing *B. thailandensis* in filtered spent *F. johnsoniae* culture. Lastly, the Kirby–Bauer assays were performed on TSA_50_ medium and not M9 glucose agar (see the Methods section). Differences in nutrient composition between TSA_50_ and M9 glucose could have affected the overall expression of and/or functionality of mechanisms to reduce the antibiotic susceptibility *F. johnsoniae *.

TolC also has a multifaceted role beyond antibiotic export. TolC has been found to be the outer-membrane protein part of type I secretion systems [55], involved in the export of siderophores [56], and an importer of toxins such as bacteriocins [57]. Furthermore, mutations that make TolC non-functional have pleiotropic consequences. For example, a gene expression analysis of a non-functional TolC mutant showed upregulation of nutrient transporters, stress response genes and central metabolism, while genes involved in nitrogen metabolism and transport were downregulated [58]. We cannot completely rule out these alternative explanations, such as nutrient acquisition, as a reason why *F. johnsoniae* could grow better in the presence of *B. thailandensis *. The Kirby–Bauer results show that there was a significant difference between monoculture evolved and coevolved lines ZOIs. This result provides credence to mutational acquisition in coevolved lines related to decreased antibiotic susceptibility. Additional functional assays would need to be performed, specifically with the *tolC* 33 bp mutant, to better understand its role in antibiotic susceptibility. In addition, further effots to isolate the bioactive compound(s) and test their effects on strains in isolation would improve MIC assays and establish mutations that lead to resistance.

A nonsynonymous mutation in *accA* in coevolved line 3 also reduced susceptibility to the *B. thailandensis *-produced antibiotic(s). In addition, this mutation guided our efforts to uncover that thailandamide was one of the antibiotics produced in *B. thailandensis* that inhibited *F. johnsoniae *. Thailandamide resistance was characterized in spontaneous mutants from *S. enterica* [36]. Wozniak and colleagues found six unique mutations for three different amino acid positions in AccA that conferred thailandamide resistance. One of these mutations was also spontaneously generated in our study (P160Q in *F. johnsoniae *, P164Q in *S. enterica *). Thailandamide is speculated to inhibit fatty acid synthesis by competitively inhibiting the binding of carboxy-biotin to acetyl-CoA carboxylase [18]. Protein modelling shows that the P164Q mutation in *S. enterica* is located in a loop region of AccA that forms the channel to the active site where carboxy-biotin binds. The authors speculate that this mutation may alter the shape of the channel, thereby limiting the accessibility of thailandamide to the active site [36].

*B. thailandensis* transposon mutants with abrogated thailandamide production effectively reduced inhibition of *F. johnsoniae *. However, we still observed inhibition with the thailandamide transposon mutants. It is likely that *F. johnsoniae* was subjected to multiple antibiotics, but other mutations conferring reduced antibiotic susceptibility in the coevolved lines did not provide insights into other *B. thailandensis *-produced antibiotics affecting *F. johnsoniae *. Futhermore, genome sequencing of *B. thailandensis* coevolved isolates did not provide insights into the *B. thailandensis *-produced antibiotic(s) affecting *F. johnsoniae *. The only possible mutation of interest was a 7 bp insertion that abrogated bactobolin production. However, a *B. thailandensis* transposon mutant with abrogated bactobolin production still inhibited *F. johnsoniae *, suggesting that there are other bioactive compounds inhibiting *F. johnsoniae *.

*F. johnsoniae* coevolved line 4 did not harbour a mutation in *tolC* or *accA*. However, the *ragB/susD* mutation was unique to coevolved line 4 and we hypothesized that this may provide an alternative mechanism for reduced antibiotic susceptibility. Some antibiotics enter bacterial cells via nutrient transporters [59, 60]. Since the *ragB/susD* rendered the protein nonfunctional, reduced antibiotic susceptibility could have been conferred to coevolved line 4 by the reduction of antibiotic uptake. We did not find this to be the case, as the *F. johnsoniae* recombinant strains with the *ragB/susD* nonsynonymous mutation had equivalent susceptibility to the *B. thailandensis *-produced antibiotic(s) as the *F. johnsoniae* ancestor. The other mutations in coevolved line 4 may reduce antibiotic susceptibility or it is possible that mutations did not fixate in the population, and we chose an isolate for sequencing that did not acquire a mutation conferring reduced antibiotic susceptibility. Bacterial population sequencing could shed light on this discrepancy.

The diversity of antibiotics [61] and corresponding resistance mechanisms [62] demonstrate the breadth of genetic novelties that have arisen from millions of years of bacterial competition. Our results provide a case study of bacterial interspecies chemical engagements that can lead to evolution in a heterogeneous environment, with insights into the complex and potentially interacting mutational paths to decreased antibiotic susceptibility.

## Supplementary Data

Supplementary material 1

Supplementary material 2

Supplementary material 3
