# Supplementary material for: A coevolution experiment between Flavobacterium johnsoniae and Burkholderia thailandensis reveals parallel mutations that reduce antibiotic susceptibility
Source: Microbiology (Reading). 2023 Feb 1;169(2):001267. doi: 10.1099/mic.0.001267 (PMC10197871; doi:10.1099/mic.0.001267)
Supplement: Supplementary material 1 [file mic-169-1267-s001.pdf]

Supplementary material for

A coevolution experiment between *Flavobacterium johnsoniae* and *Burkholderia thailandensis* reveals parallel mutations that reduce antibiotic susceptibility

John L. Chodkowski[1] and Ashley Shade[1,2,3*]

[1]Department of Microbiology and Molecular Genetics, Michigan State University, East Lansing, MI 48824, USA

[2]Department of Plant, Soil and Microbial Sciences, Michigan State University, East Lansing, MI 48824, USA

[3]Program in Ecology, Evolution and Behavior, Michigan State University, East Lansing, MI 48824, USA

* Corresponding author and material requests. Email: shadeash@msu.edu; ORCiD: 0000-0002-7189-3067

Data and code availability: https://github.com/ShadeLab/Paper_Chodkowski_Coevolution_2022

Supplementary Tables

Supplementary Table 1 Summary of *tolC* loci in *F. johnsoniae*

Locus	Protein ID	Top blastp hit	AA length
FJOH_RS06580	WP_012023347.1	TolC family protein	444
FJOH_RS07030	WP_012023433.1	“	451
FJOH_RS08665	WP_012023747.1	“	461
FJOH_RS14165	WP_012024794.1	“	415
FJOH_RS15250	WP_012025000.1	“	436
FJOH_RS15955	WP_008463753.1	“	415
FJOH_RS16725	WP_012025212.1	“	469
FJOH_RS16800	WP_012025225.1	“	484
FJOH_RS17335	WP_044047818.1	“	426
FJOH_RS20485	WP_012025935.1	“	472
FJOH_RS22150	WP_044048008.1	“	461
FJOH_RS22200	WP_012026267.1	“	412
FJOH_RS22240	WP_012026275.1	“	472
FJOH_RS23175	WP_012026451.1	“	417
FJOH_RS25120	WP_012026826.1	“	479
FJOH_RS25325	WP_012026867.1	“	439

Supplementary Table 2 Percent identity matrix for all TolC proteins annotated in *F. johnsoniae*. Mutations in coevolved isolates were found in RS06580. Multiple sequence alignments were made using Clustal Omega

RS06580	100															
RS15250	24.2	100														
RS20485	15.99	15.5	100													
RS15955	13.94	11.86	13.51	100												
RS16800	16.46	15.21	14.84	16.19	100											
RS25120	15.15	14.65	12.29	18.49	31.99	100										
RS16725	19.07	18.78	11.62	18.09	32.03	29.68	100									
RS22240	15.46	15.08	13.89	16.71	28.6	34.48	40.51	100								
RS17335	17.71	19.84	14.68	16.48	15.38	13.95	15.61	15.3	100							
RS22150	18.02	17.5	15.11	11.83	14.15	15.82	15.92	15.42	16.67	100						
RS23175	16.84	15.3	13.35	14.48	18.53	17.26	15.84	15.32	15.95	13.57	100					
RS14165	17.36	14.82	13.66	16.49	17.05	18.09	14.51	14.78	13.06	18.25	23.08	100				
RS22200	17.05	17.69	12.37	17.21	15.37	14.11	14.8	15.82	18.68	15.17	22.19	28.1	100			
RS07030	20.15	18.72	14.77	14.66	14.63	14.11	14.5	14	17.95	16.1	15.38	12.94	14.21	100		
RS25325	20.54	19.55	15.84	12.87	13.51	13.51	16.12	15.62	12.5	21.22	16.71	15.4	14.86	24.65	100	
RS08665	22	21.84	17.36	14.33	14.47	16.2	19.01	16.41	18.59	18.76	15.03	18.16	18.59	20.15	20.35	100

Supplementary Table 3 Primers used in this study

Primer	Sequence (5' > 3')	Description
1001	TTGCTTATTTGGGAGGAACAACA	Used to amplify tolC for nested PCR round 1
1002	CATCTGCTTTTGCAGCGATGA	Used to amplify tolC for nested PCR round 1
1003	GCTAGTCTAGAGCATCAGTTGAGTTTTCACTGGA	Used for nested PCR round 2 to construct pJC101 and pJC102; XbaI site underlined
1004	GCTAGGGATCCAAGCTTGCAACCTGGCTTTC	Used for nested PCR round 2 to construct pJC101 and pJC102; BamHI site underlined
1005	AAATGACGGTCCCATCTCAAA	Used to amplify tolC to confirm successful mutant construction
1006	CCCATGTAAAACTTCAATGCGT	Used to amplify tolC to confirm successful mutant construction
1010	TGAGAACCAAAGGCTGGGAA	Used to amplify ragB/susD for nested PCR round 1
1011	GGTACATTGTTTTCGGCGCA	Used to amplify ragB/susD for nested PCR round 1
1012	GCTAGTCTAGATGGGGATTAACCAGCGACAG	Used for nested PCR round 2 to construct pJC103; XbaI site underlined
1013	GCTAGGGATCCTTCACCTGCATCGGCAGTTC	Used for nested PCR round 2 to construct pJC103; BamHI site underlined
1014	ATGCTCCCGCAAAACCAAGA	Used to amplify ragB/susD to confirm successful mutant construction
1015	ATCAGGACCAGTTGTTGCCG	Used to amplify ragB/susD to confirm successful mutant construction

Supplementary Table 4 PCR conditions for nested PCR round 1

Reagent	Volume (μL)
Template (6.25 ng/μL)	10
Forward/Reverse primers (10 μM)	2.5
10 mM dNTPs (Sigma-Aldrich, St. Louis, MO)	1
Phusion DNA polymerase (New England BioLabs, Ipswich, MA)	0.5
Phusion 5X buffer (HF buffer for *tolC* and GC buffer for *ragB/susD*)	9.5
Nuclease-free water	24

Supplementary Table 5 PCR conditions for nested PCR round 2

Reagent	Volume (μL)
Template (1 ng/μL; PCR product from R1)	0.5
Forward/Reverse primers (10 μM)	2.5
10 mM dNTPs	1
Phusion DNA polymerase	0.5
Phusion 5X buffer (HF buffer for *tolC* and GC buffer for *ragB/susD*)	9.5
Nuclease-free water	33.5

Supplementary Table 6 Reagents and reaction volumes for restriction enzyme digestion

Reagent	Volume (μL)
Nested PCR R2 products or pYT354 (1 μg/μL)	1
10X cutsmart buffer (New England BioLabs, Ipswich, MA)	5
BamHI-HF (New England BioLabs, Ipswich, MA)	1 (20 units)
XbaI (New England BioLabs, Ipswich, MA)	1 (20 units)
Nuclease-free water	42

Supplementary Table 7 Reagents and reaction volumes/mass for ligation reactions

Reagent	Volume/Mass
Insert (~3.2 for *tolC*, ~3.1 kbp for *ragB/susD*)	Varied[a]
Vector (~7.7 kbp)	50 ng
T4 DNA ligase (New England BioLabs, Ipswich, MA)	1 μL
10 X T4 DNA ligase buffer (New England BioLabs, Ipswich, MA)	2 μL
Nuclease-free water	Up to 20 μL

[a]To achieve a 1:3 vector:insert molar ratio, 61.49 ng was used from *tolC*-containing PCR products and 59.37 ng used from *ragB/susD*-containing PCR products.

Supplementary Figures

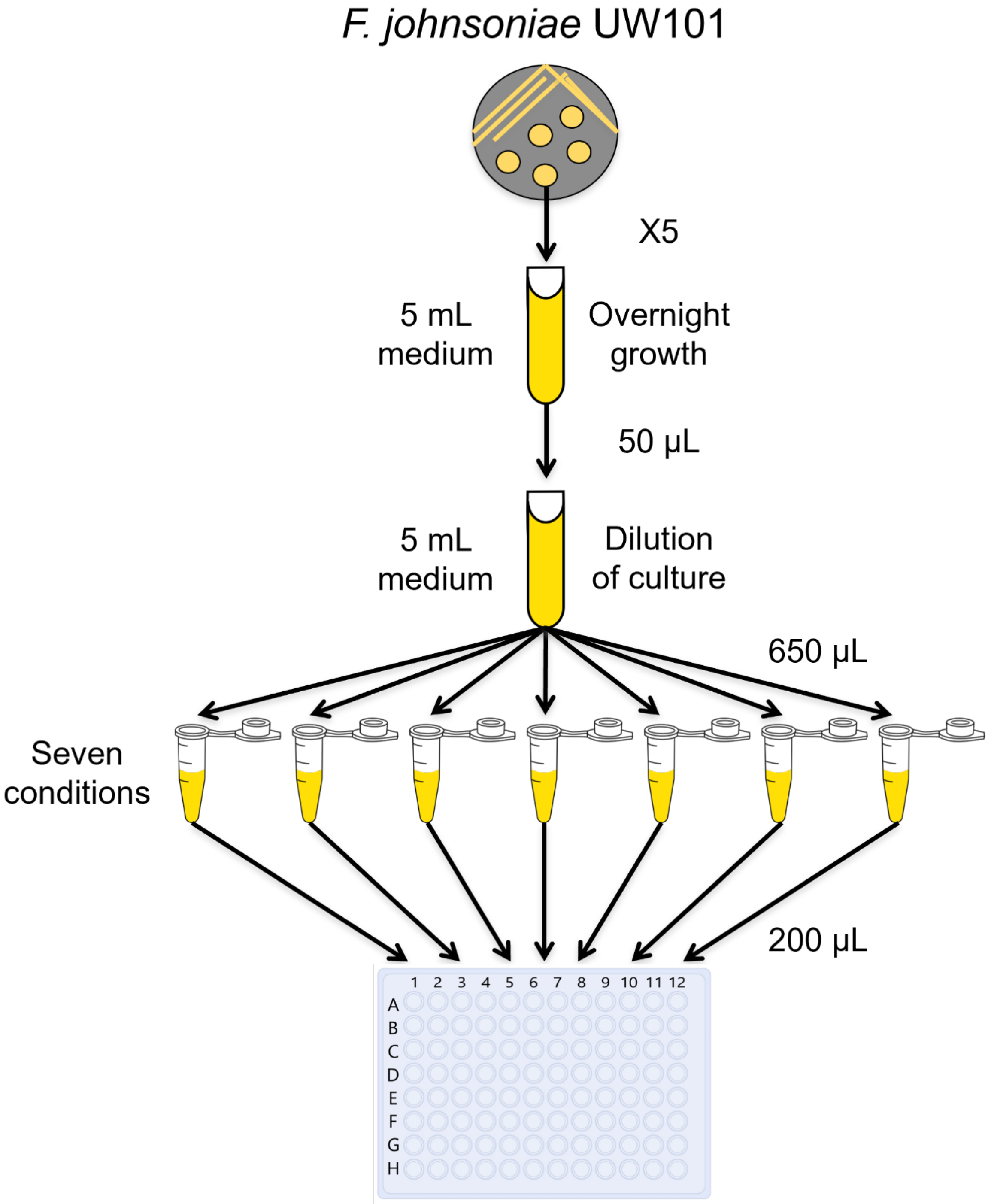

Supplementary Fig. 1 Schematic of preparation for efflux pump inhibitor experiment.

Passage | *B. thailandensis* | *F. johnsoniae*

1

2

3

4

5

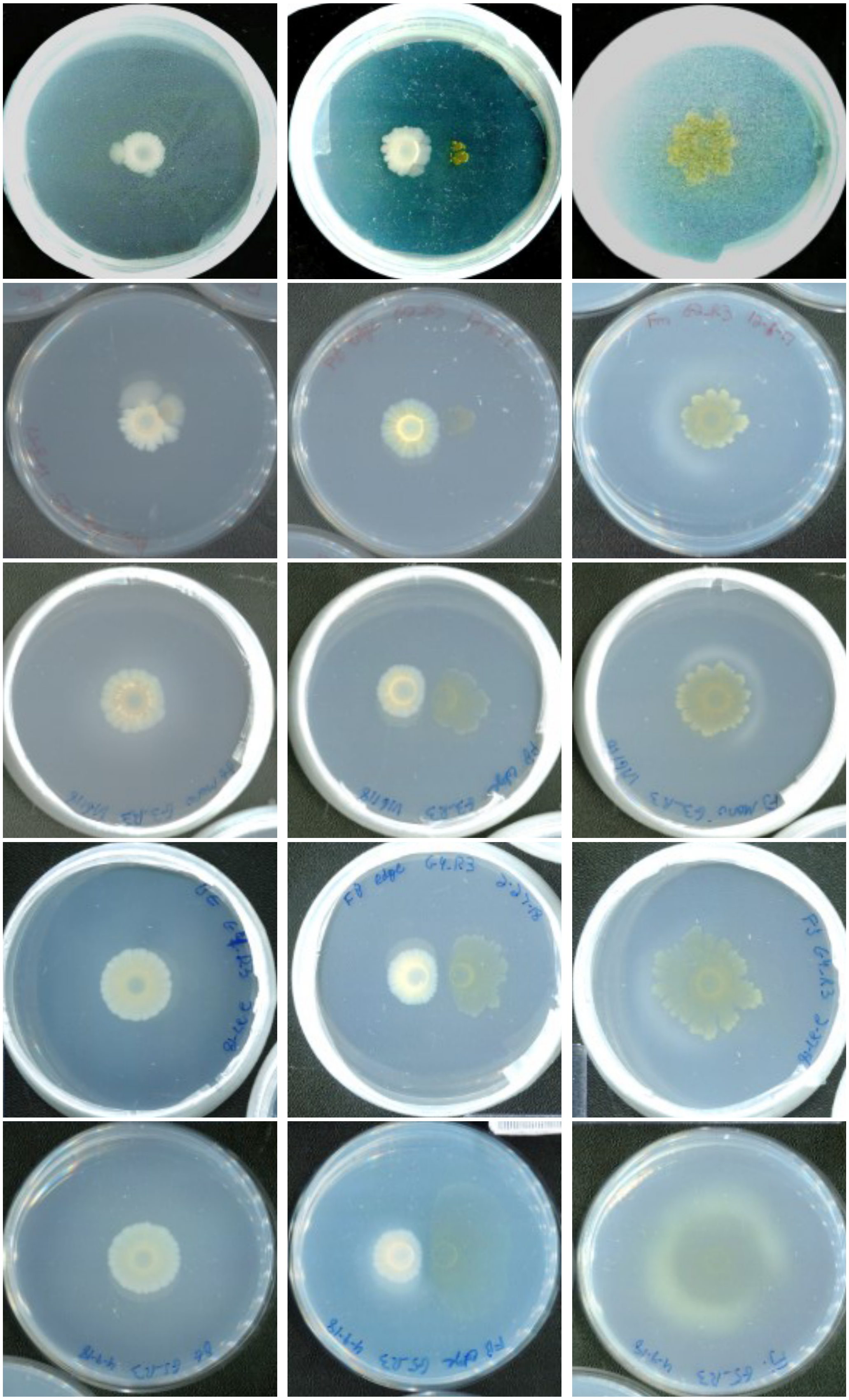

44

Supplementary Fig. 2 Colony morphologies and growth success over the (co)evolution experiment. Plate images were taken at 1.5 months after each plate passage. Shown are colony morphologies and growth success of *B. thailandensis* monoculture (column 1), co-plated *B. thailandensis-F. johnsoniae* (column 2), and *F. johnsoniae* monoculture (column 3) for a representative independent replicate (rep 3). Each row is plate passage

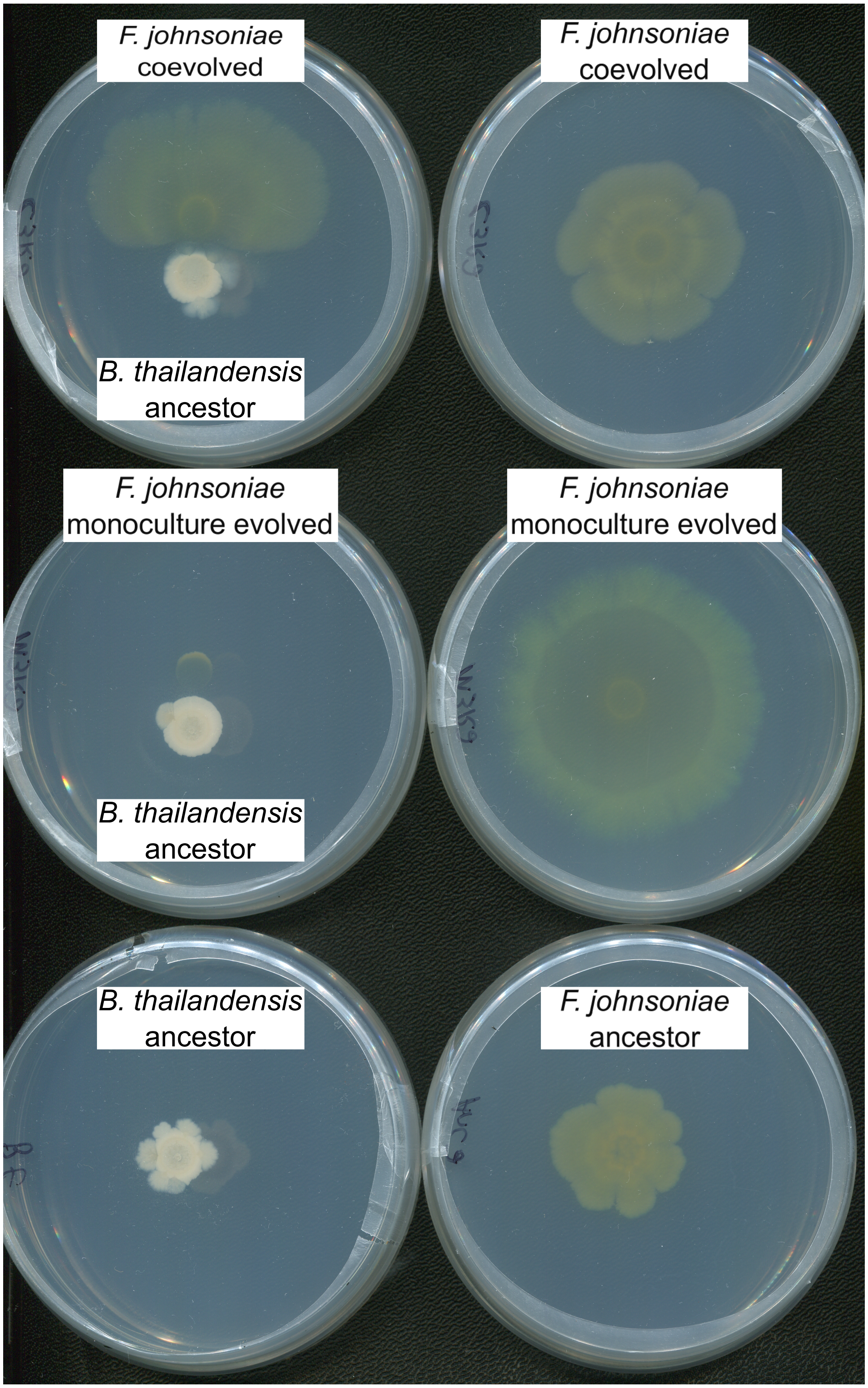
F. johnsoniae coevolved
F. johnsoniae coevolved
B. thailandensis ancestor
F. johnsoniae monoculture evolved
F. johnsoniae monoculture evolved
B. thailandensis ancestor
B. thailandensis ancestor
F. johnsoniae ancestor

Supplementary Fig. 3 Coevolved *F. johnsoniae* has reduced susceptibility to a *B. thailandensis*-produced antibiotic(s). Coevolved *F. johnsoniae* can grow better in the presence of *B. thailandensis* (column 1, top row) compared to the monoculture evolved *F. johnsoniae* (column 1, middle row). Monocultures are shown as a growth control (column 2, top and middle rows). Shown are evolved lines from one of the independent replicates (rep 3) from the fifth plate passage. Ancestor *F. johnsoniae* and ancestor *B. thailandensis* are shown as additional monoculture controls (bottom row). Images were taken after incubation for 1.5 months

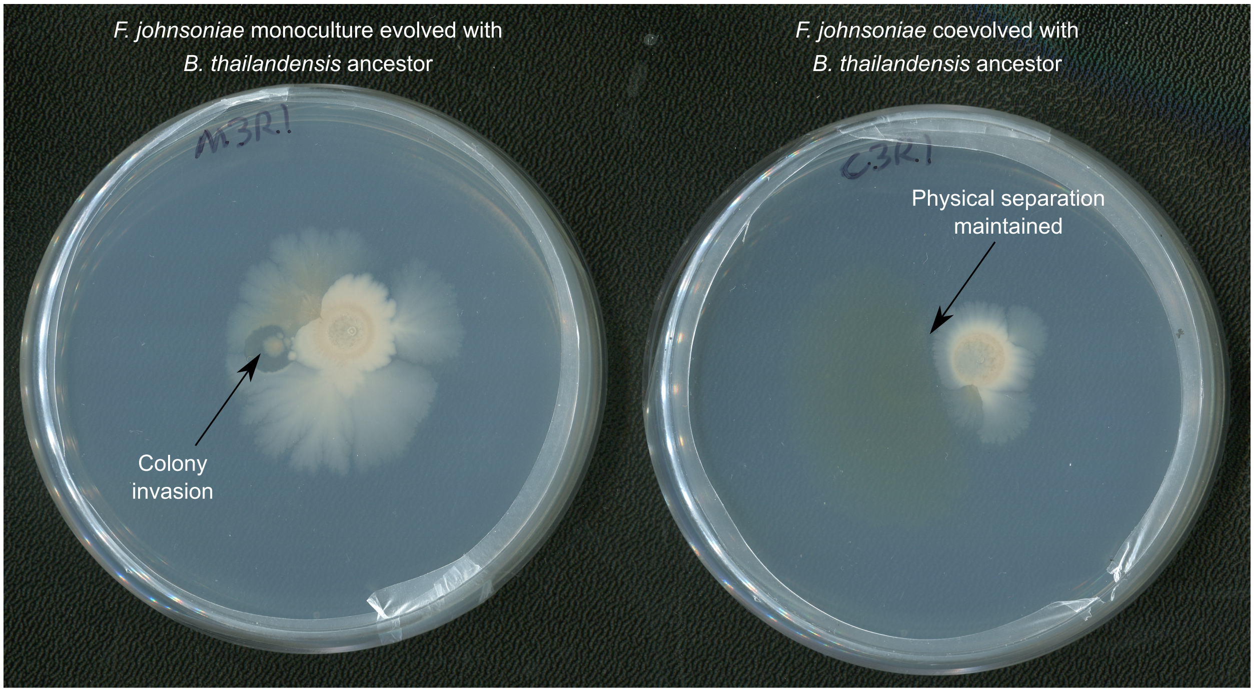

Supplementary Fig. 4 Coevolved *F. johnsoniae* can resist colony invasion. On each plate, *F. johnsoniae* is on the left and *B. thailandensis* (right) is on the right. The *B. thailandensis* ancestor was co-plated with *F. johnsoniae* evolved monoculture (left plate) and *F. johnsoniae* coevolved (right plate) from the fifth plate passage. Plates were incubated for 2.5 months to allow the chance for physical interactions to occur

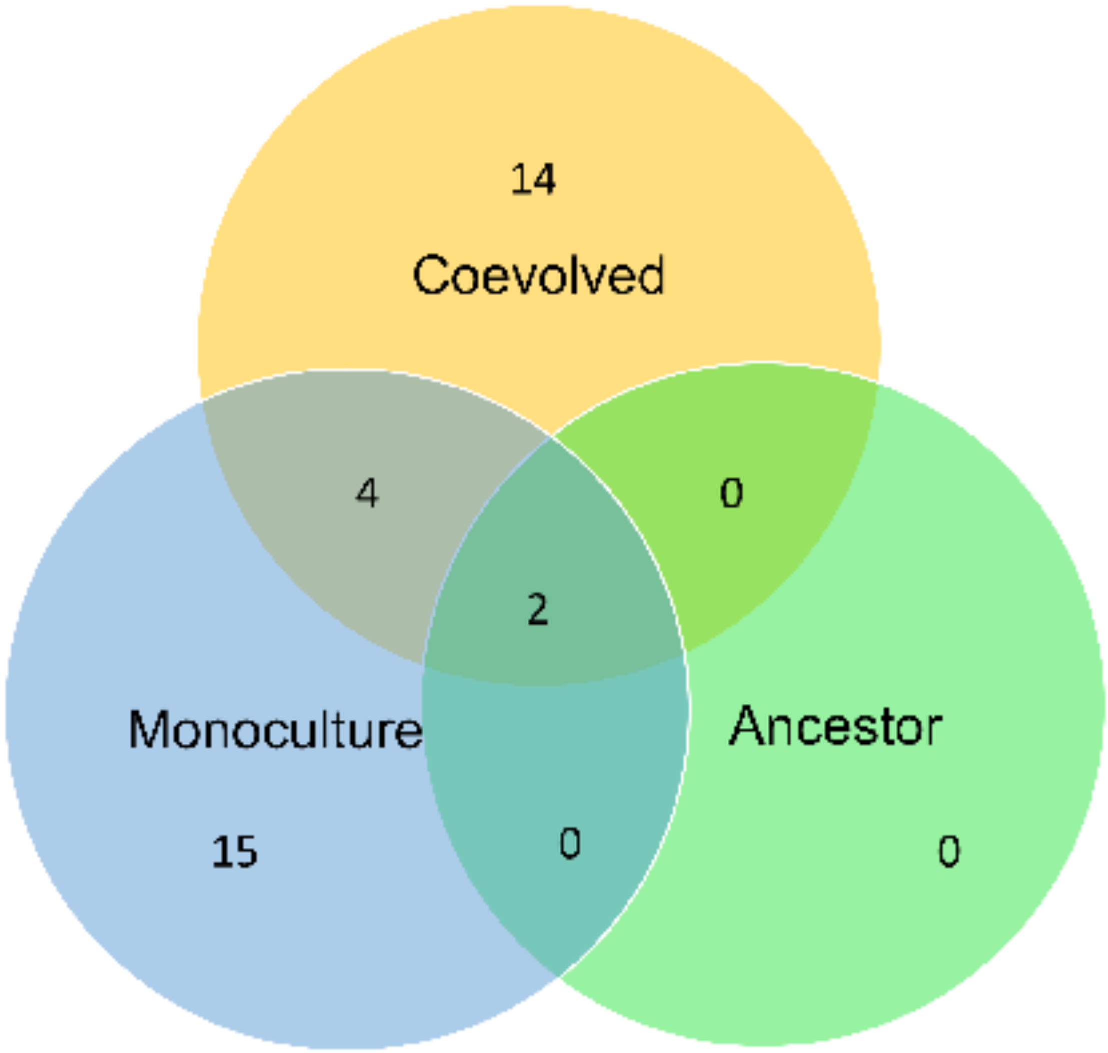

Supplementary Fig. 5 Coevolved lines acquire unique mutations as a result of interspecies interactions. Shown is a Venn diagram comparing distinctions and overlaps of gene loci where mutations were observed in the ancestor, monoculture evolved lines, and coculture evolved lines

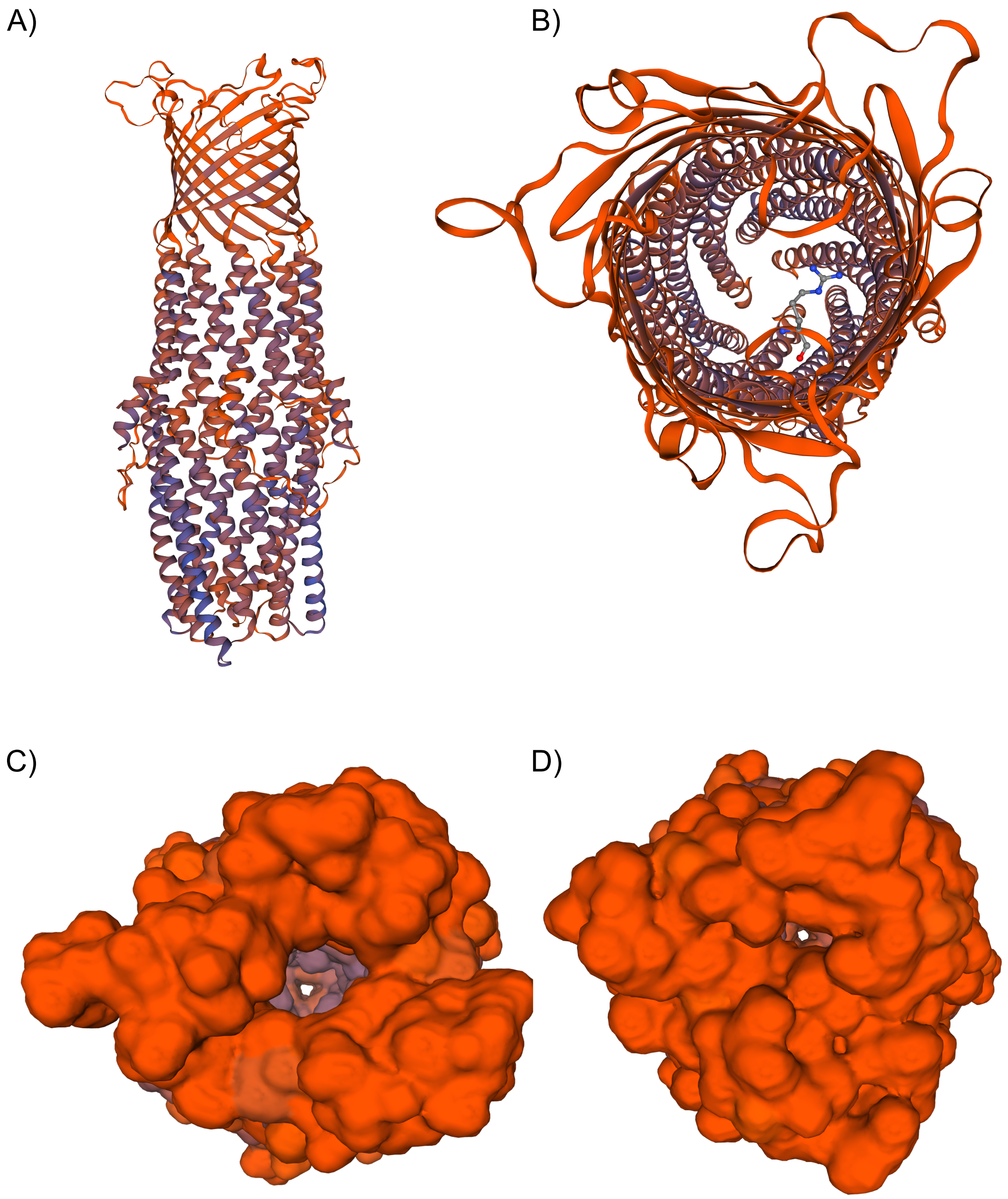

Supplementary Fig. 6 A nonsynonymous mutation in TolC narrows the opening of the efflux channel. A model of TolC (A) with the G83R nonsynonymous mutation. TolC is rotated +90 about the x-axis in panels B-D such that TolC is viewed from top looking down the channel. The G83R residue (B) is located on one of the extracellular loops of TolC. The opening of the efflux channel in WT TolC (C) is predicted to narrow due to the G83R mutation (D). TolC from *E. coli* was used as the template (SMTL ID: 6wxi.1) to construct the target *F. johnsoniae* WT TolC (SWISS-MODEL: GMQE=0.6, Seq ID=19.06) and G83R TolC (SWISS-MODEL: GMQE= 0.59, Seq ID=19.06) models.

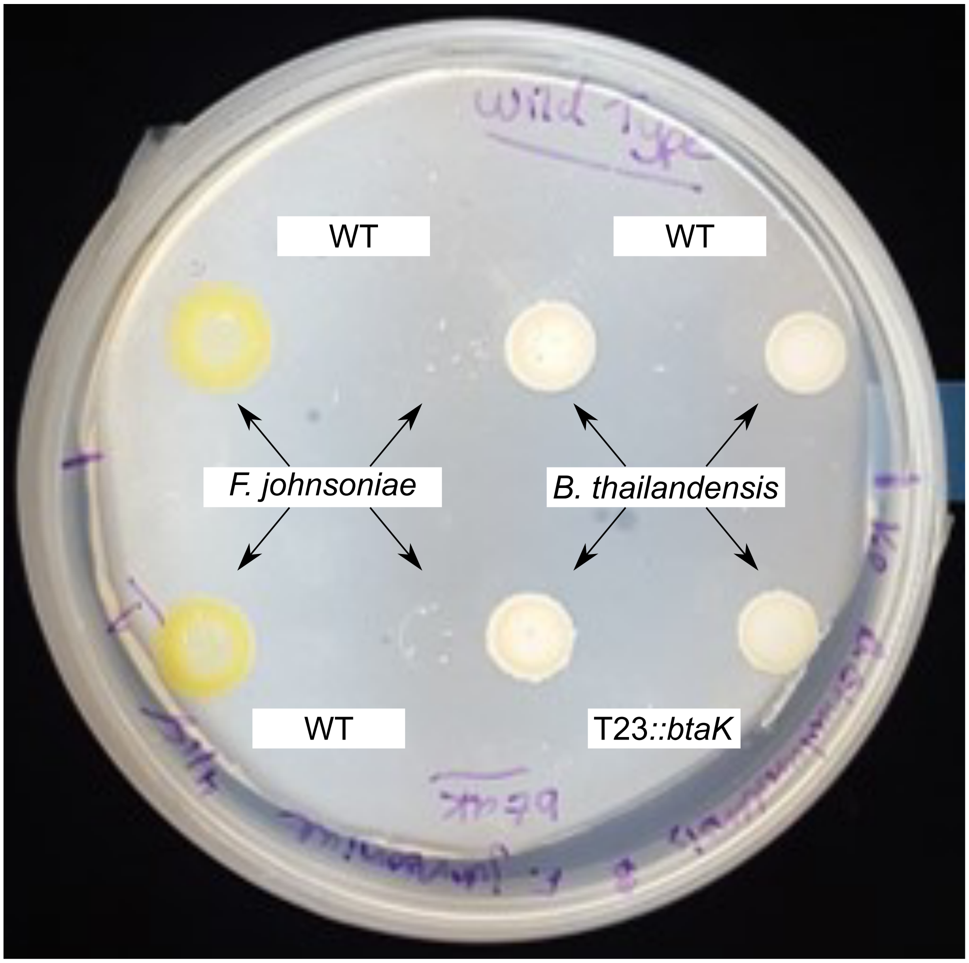

Supplementary Fig. 7 A *B. thailandensis* btaK::T23 mutant with abrogated bactobolin production still inhibits *F. johnsoniae*. *B. thailandensis* WT (top) and *B. thailandensis* btaK::T23 (bottom) was co-plated with *F. johnsoniae* WT. Strains were also plated outside the interspecies interaction zone as controls

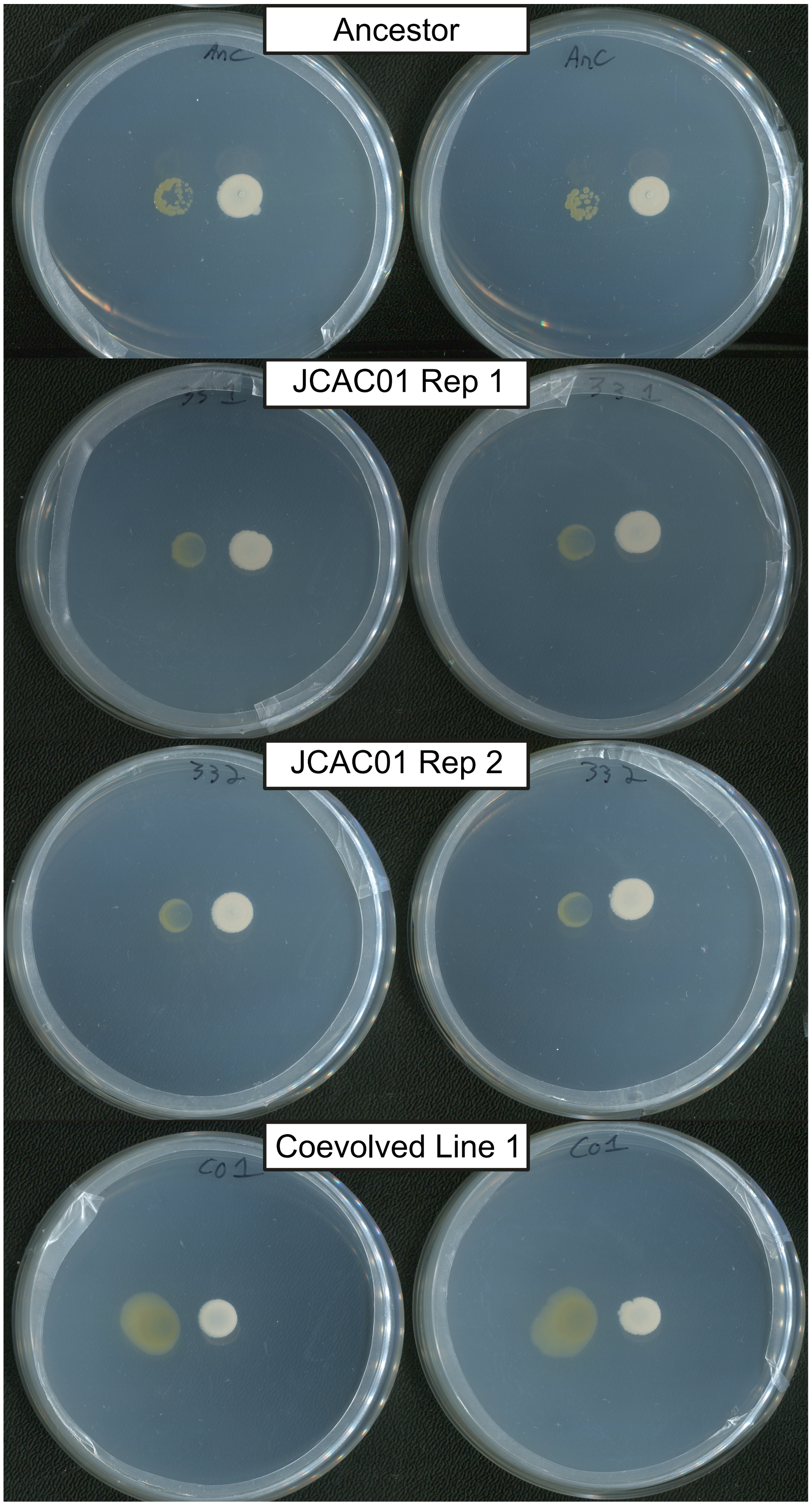

Supplementary Fig. 8 *F. johnsoniae* recombinants display a reduction in antibiotic susceptibility, but not to the same degree observed in coevolved lines. The 33 bp deletion in FJOH_RS06580 was placed into the *F. johnsoniae* ancestor and co-plated with *B. thailandensis*. Two confirmed successful recombinants (reΔ33_*tolC* , replicates 1&2)

are less inhibited by *B. thailandensis* compared to the *F. johnsoniae* ancestor but are more inhibited compared to the coevolved line from which FJOH_RS06580 was amplified to create the recombinants. All strains were co-plated with the *B. thailandensis* ancestor. Plates were imaged after a month of incubation

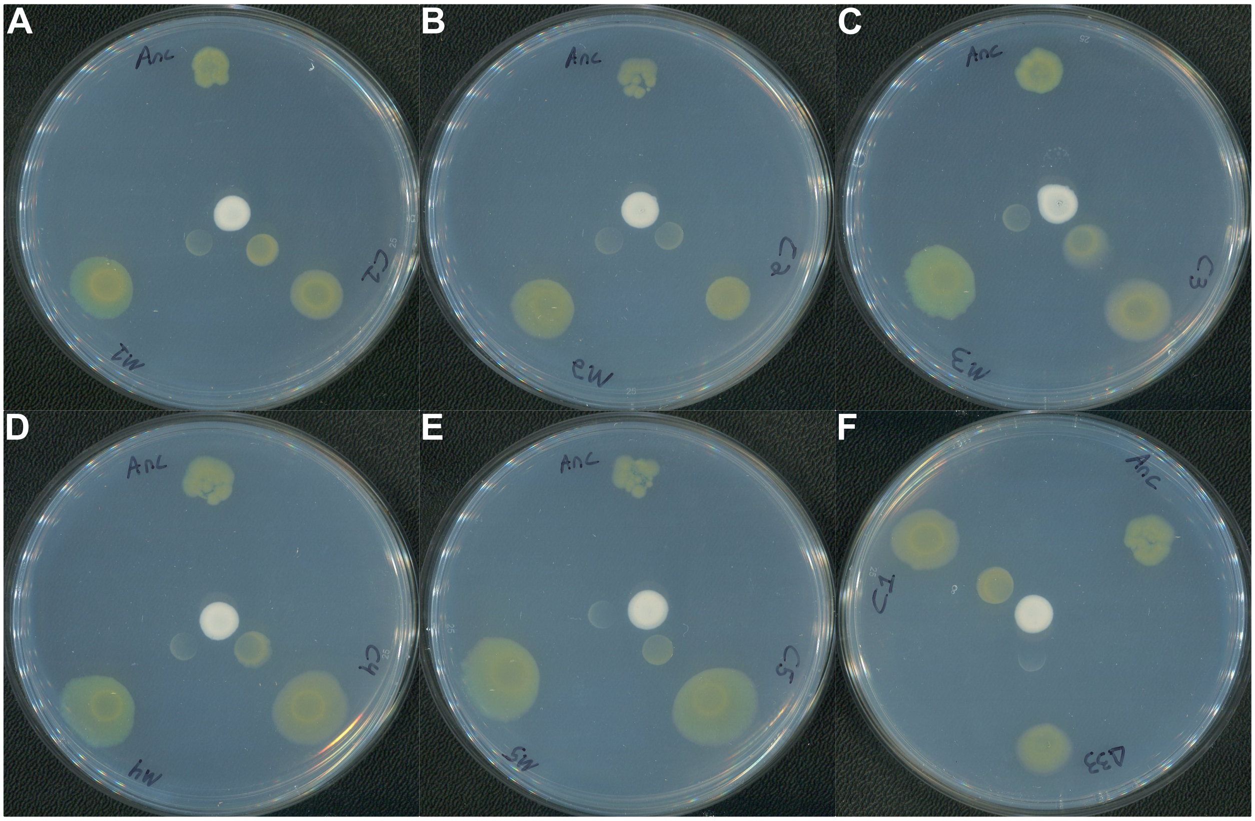

Supplementary Fig. 9 Growth success of *F. johnsoniae* strains when co-plated with *B. thailandensis*. *F. johnsoniae* strains (yellow) were plated in the vicinity of the *B. thailandensis* ancestor (beige, middle colony) to observe growth inhibition. In each panel, the *F. johnsoniae* ancestor (Anc; panels A-F), the *F. johnsoniae* coevolved replicates from the 5th plate passage (C1, panels A-F; C2-C5, panels A-E), the *F. john*soniae evolved monocultures from the 5th plate passage (M1-M5, panels A-E), and the *F. johnsoniae* recombinant strain, reΔ33_*tolC* (Δ33, panel F), were plated in a triangular formation around *B. thailandensis*. Duplicate colonies were spotted at the exterior of the plates as a growth control. Plates were imaged after a week of incubation
